# NISNet3D: three-dimensional nuclear synthesis and instance segmentation for fluorescence microscopy images

**DOI:** 10.1038/s41598-023-36243-9

**Published:** 2023-06-12

**Authors:** Liming Wu, Alain Chen, Paul Salama, Seth Winfree, Kenneth W. Dunn, Edward J. Delp

**Affiliations:** 1 Video and Image Processing Laboratory, School of Electrical and Computer Engineering, Purdue University, West Lafayette, IN 47907 USA; 2 Department of Electrical and Computer Engineering, Indiana University-Purdue University Indianapolis, Indianapolis, IN 46202 USA; 3 Department of Pathology and Microbiology, University of Nebraska Medical Center, Omaha, NE 68198 USA; 4 School of Medicine, Indiana University, Indianapolis, IN 46202 USA

**Keywords:** Biological techniques, Computational biology and bioinformatics

## Abstract

The primary step in tissue cytometry is the automated distinction of individual cells (segmentation). Since cell borders are seldom labeled, cells are generally segmented by their nuclei. While tools have been developed for segmenting nuclei in two dimensions, segmentation of nuclei in three-dimensional volumes remains a challenging task. The lack of effective methods for three-dimensional segmentation represents a bottleneck in the realization of the potential of tissue cytometry, particularly as methods of tissue clearing present the opportunity to characterize entire organs. Methods based on deep learning have shown enormous promise, but their implementation is hampered by the need for large amounts of manually annotated training data. In this paper, we describe 3D Nuclei Instance Segmentation Network (NISNet3D) that directly segments 3D volumes through the use of a modified 3D U-Net, 3D marker-controlled watershed transform, and a nuclei instance segmentation system for separating touching nuclei. NISNet3D is unique in that it provides accurate segmentation of even challenging image volumes using a network trained on large amounts of synthetic nuclei derived from relatively few annotated volumes, or on synthetic data obtained without annotated volumes. We present a quantitative comparison of results obtained from NISNet3D with results obtained from a variety of existing nuclei segmentation techniques. We also examine the performance of the methods when no ground truth is available and only synthetic volumes were used for training.

## Introduction

Over the past 10 years, various technological developments have provided biologists with the ability to collect 3D microscopy volumes of enormous scale and complexity. Methods of tissue clearing combined with automated confocal or lightsheet microscopes have enabled three-dimensional imaging of entire organs or even entire organisms at subcellular resolution. Multiplexing methods have been developed so that one can now simultaneously characterize 50 or more targets in the same tissue. However, as biologists analyze these large volumes (tissue cytometry), they quickly discover that the methods of automated image analysis necessary for extracting quantitative data from volumes of this scale are frequently inadequate for the task. In particular, while effective methods for distinguishing (segmenting) individual cells are available for analyses of two-dimensional images, corresponding methods for segmenting cells in three-dimensional volumes are generally lacking. The problem of three-dimensional image segmentation thus represents a bottleneck in the full realization of 3D tissue cytometry as a tool in biological microscopy.

There are generally two typical categories of approaches for segmentation: non-machine learning based image processing and computer vision techniques and techniques based on machine learning and in particular deep learning^1,2^. The traditional image processing techniques (e.g. watershed transform, thresholding, edge detection, and morphological operations) can be effective on one type of microscopy volume but may not generalize to other types of volumes without careful parameter tuning. Segmentation techniques based on deep learning have shown great promise, in some cases providing accurate and robust results across a range of image types^3–7^.

The utility of deep learning methods is limited by the large amount of manually annotated data (note: we use the terms “ground truth”, “manual annotations”, and “hand annotated volumes” interchangeably in this paper) needed for training and validation. Annotation is a labor-intensive and time-consuming process, especially for a 3D volume. While tools have been developed to facilitate the laborious process of manual annotation^8–11^, the generation of training data for 3D microscopy images remains a major obstacle to implementing 3D segmentation approaches based upon deep learning.

The problem of generating sufficient training data can be alleviated using data augmentation, a process in which existing manually annotated training data is supplemented with synthetic data generated from modifications of the manually annotated data^12–15^. An alternative method is to use synthetic data for training^14,16,17^. One approach for generating synthetic 3D fluorescent microscopy volumes is by stacking 2D synthetic image slices using the 2D distributions of the fluorescent markers and the use of Generative Adversarial Networks (GANs)^18,19^.

Convolutional neural networks (CNNs) have had great success for solving problems such as object classification, detection, and segmentation^20,21^. The encoder-decoder architecture has been widely used for biomedical image analysis including volumetric segmentation^22–24^, medical image registration^25^, nuclear segmentation^3,6,15,26–32^, and 2D cell nuclei tracking^33,34^. However, most CNNs are designed for segmenting two-dimensional images and cannot be directly used for segmentation of 3D volumes^26,29,35^. Other methods process images slice by slice and fuse together two dimensional results to form 3D segmentation results that fail to consider the 3D anisotropy of microscopy volumes^3,14,27^.

True 3D segmentation is important for the analysis of biological structures. Nearly all biological structures require 3D characterization. The most common method for generating 3D images of biological structures is based upon collection of a sequence of 2D images. The axial dimension is sampled at a rate sufficient so that 3D structures are captured in the stack of images. Ideally the sampling rate should at the level of the resolution of the microscopy technique. While 3D microscopy is based upon volumes assembled from series of 2D images, it is important to appreciate that sequential images collected from a 3D volume differ from 2D images collected from a single plane in that sequential images collected from a 3D volume are not independent of one another, and thus contain information that is unique to the axial dimension. Insofar as methods of 3D segmentation recognize that the nature of object boundaries in 3D microscopy images differ in the axial dimension from those in the lateral dimension, the use of this axial information will support more accurate segmentation in the axial dimension.

In this paper, we are interested in segmenting nuclei in 3D microscopy volumes. There are two aspects of segmentation: semantic segmentation and instance segmentation. Semantic segmentation treats multiple objects within one general category as a single entity^36^; voxels in a volume are indicated as nuclei or not. Instance segmentation identifies objects as different entities. In other words, while semantic segmentation is widely used for separating foreground objects and background structures in an image, instance segmentation is capable of distinguishing individual objects that may overlap one another in an image^36^. Here we are interested in instance segmentation using deep learning so that we can analyze nuclei whose images overlap with one another. We describe here 3D Nuclei Instance Segmentation Network (NISNet3D), a deep learning-based 3D instance segmentation technique.

NISNet3D is a true 3D instance segmentation method that operates directly on 3D volumes, using 3D CNNs to exploit 3D information in a microscopy volume, thereby generating more accurate segmentations of nuclei in 3D image volumes. It can be trained on both actual microscopy volumes and synthetic microscopy volumes or a combination of both. NISNet3D can also be trained on synthetic data and further lightly retrained on limited number of other types of microscopy data as an incremental improvement.

## Results

### NISNet3D—general approach

Nuclei instance segmentation methods typically include a foreground and background separation step and a nuclei instance identification and segmentation step. Non-machine learning methods use thresholding such as Otsu’s thresholding for separating background and foreground and use a watershed transformation to identify individual cell nuclei. However, thresholding methods are sensitive to variability in intensity and may not generate accurate segmentation masks. Also, watershed transform segmentation without accurate markers may generate under-segmentation or over-segmentation results. Machine learning methods typically generate more accurate results if they are provided with enough training data. However, most current methods that are designed for nuclei instance segmentation only work with 2D images or use a 2D to 3D construction, which may not fully capture the 3D spatial information.

**Figure 1:**
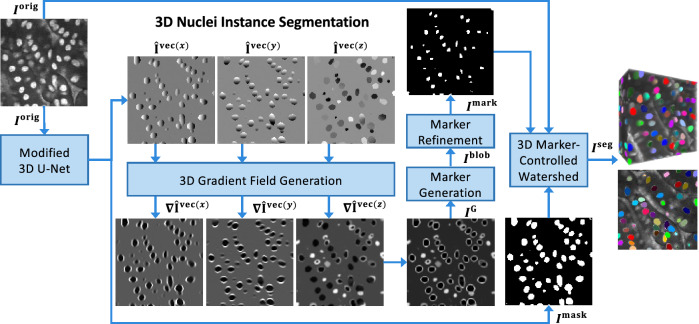
Overview of NISNet3D for 3D nuclei instance segmentation. NISNet3D uses a modified 3D U-Net for nuclei segmentation and 3D vector field array estimation where each voxel represents a 3D vector pointing to the nearest nuclei centroid. NISNet3D then generates a 3D gradient field array from the 3D vector field array and further generates refined markers. Finally, a 3D watershed transform segmentation operates on three inputs (the seeds from the gradients, the binary segmentation masks, and the original microscopy volume) to generate the instance segmentation volume.

NISNet3D uses a modified 3D U-Net with attention and residual blocks, a 3D marker-controlled watershed transform, and a nuclei instance segmentation system for separating touching nuclei. NISNet3D also uses an improved distance transform for marker-controlled the watershed transform by generating a 3D gradient volume to obtain more accurate markers for each nuclei. The overall approach of NISNet3D is described here (and shown in Fig. 1). A complete, detailed description of NISNet3D and all methods used here are provided in “Methods”.

3D U-Net was chosen as the base architecture since 2D U-Nets have shown to been effective in many biomedical applications^22^. While there are existing 3D techniques including a 3D U-Net architecture^23^, these methods do not exploit the true 3D information in the microscopy volume, address the issue of overlapping nuclei, nor are they designed to analyze large microscopy volumes. The instance segmentation built into NISNet3D generates more accurate markers than the direct distance transform used in the typical watershed transform by learning a 3D vector field array which contains information used to accurately estimate the location of nuclei centroids and boundaries. As shown in Fig. 1, using the 3D vector field array, NISNet3D generates a 3D gradient array to aid in the detection of nuclei boundaries. This approach also refines the 3D markers that are used by the watershed transform for segmentation. Our hypothesis is that the 3D gradient array along the boundaries of touching nuclei should be very large and by extending 3D U-Net’s semantic segmentation using the proposed vector field array, NISNet3D will achieve better instance segmentation.

### Quantitative analysis of segmentation quality

**Table 1:** The description of the five datasets used for evaluation.

Original microscopy volumes								
Volume ID	Volume description	Original size (${X}\times {Y}$ $\times {Z}$)	Subvolume size (${X}\times {Y}$ $\times {Z}$)	Number of subvolumes in volume	Number of annotated subvolumes	Percentage of volume annotated (%)	Number of subvolumes generated	Percentage of volume generated (%)
${\mathcal {V}}_1$	Scale^37^-cleared rat kidney	$512\times 512 \times 200$	$128\times 128 \times 64$	50	1	2	250	500
${\mathcal {V}}_2$	Shallow rat liver	$512\times 512 \times 32$	$128\times 128 \times 32$	16	16	100	250	1562
${\mathcal {V}}_3$	BABB^38^-cleared rat kidney	$512\times 512 \times 415$	$128\times 128 \times 64$	104	9	8.6	250	240
${\mathcal {V}}_4$	Cleared mouse intestine	$512\times 930 \times 157$	$128\times 128 \times 40$	114	5	4.3	200	175
${\mathcal {V}}_5$	Zebrafish brain	$2000\times 1450 \times 397$	$64\times 64 \times 64$	4392	27	0.6	n/a	n/a

Segmentation accuracy in selected subvolumes of each microscopy volume was evaluated by comparison with results obtained by manual annotation with ITK-SNAP^8^, which serve as ground truth^39^. Each subvolume was annotated by a single annotator, and each annotation was examined and approved by a biologist (co-author Dunn). We have annotated 1, 16, 9, 5 subvolumes for ${\mathcal {V}}_1$–${\mathcal {V}}_4$, respectively. See Table 1 for the size of each annotated subvolume. Datasets ${\mathcal {V}}_1$–${\mathcal {V}}_4$ and corresponding ground truth subvolumes are available for download^39^. Detailed information of all original five datasets used in our evaluation is shown in Table 1.

### Generation of synthetic training data

Deep learning methods generally require large amounts of training samples. Manually annotating ground truth is a tedious task, particularly for 3D microscopy volumes. To address this issue, we generated synthetic microscopy volumes for training NISNet3D.

**Figure 2:**
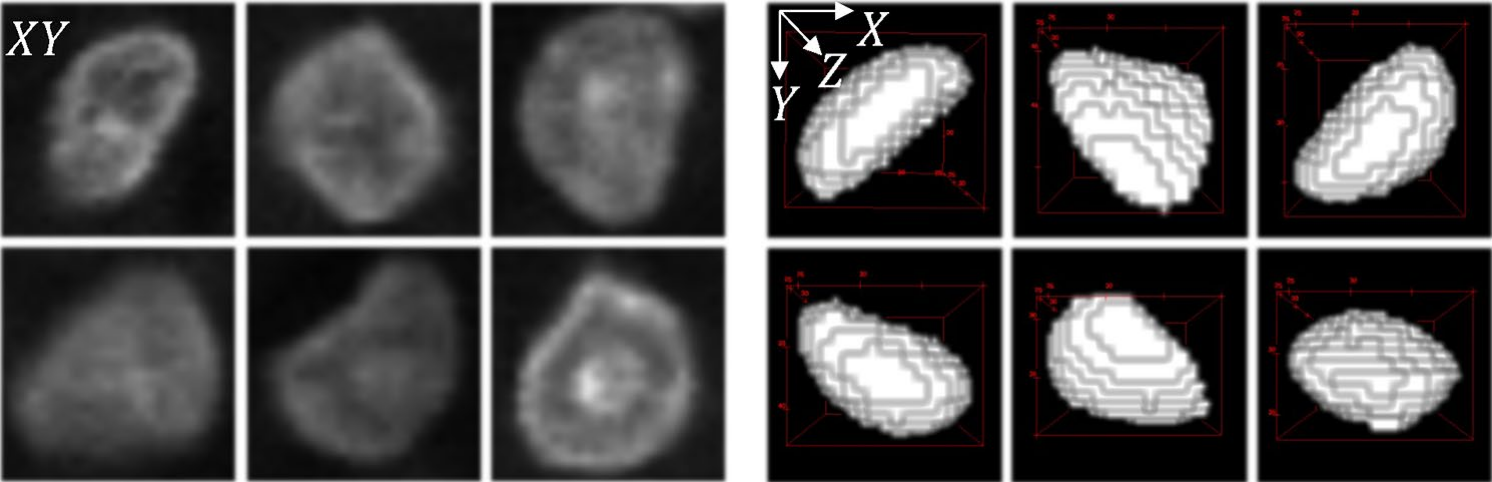
Generating synthetic nuclei from deformed ellipsoids. The three columns on the left show the *XY* focal planes of the non-ellipsoidal shaped nuclei in actual microscopy volumes. The voxel resolutions are $1\times 1\times 1\, \textrm{micron}^3\, (X\times Y\times Z)$. The columns on the right show 3D renderings of various nuclei from a synthetic binary nuclei segmentation volume after using elastic deformation.

To generate synthetic microscopy volumes, we first generate synthetic segmentation masks, which will be used as the synthetic ground truth masks during training, by iteratively adding initial binary nuclei to an empty 3D volume. Although nuclei are generally ellipsoidal, many cell types are characterized by nuclei of different shapes, which can be approximated as deformed ellipsoids (Fig. 2), we model these initial nuclei as deformed 3D binary ellipsoids having random sizes and orientations. Specifically, we use an elastic transformation^40^ to deform the 3D binary masks of the nuclei. Details of the procedures for generating initial binary segmentation maps and deforming them are described in “ Synthetic segmentation mask generation”. Examples of deformed ellipsoids are shown in Fig. 2.

We modify our synthetic microscopy volume generation method SpCycleGAN^6,7,28^ to model non-ellipsoidal or irregularly shaped nuclei. We have shown in our previous work^7,41–43^ that SpCycleGAN can be used to generate synthetic microscopy volumes that can be used for training. Thus, we used the unpaired image-to-image translation model known as SpCycleGAN^7^ for generating synthetic microscopy volumes. By unpaired we mean that the binary segmentation masks we created above are not the ground truth of actual microscopy images. SpCycleGAN is a variation of CycleGAN^44^ that better maintains the spatial relationship of the nuclei (see “Synthetic microscopy volume generation”). Following training SpCycleGAN, we can generate synthetic 3D volumes with known ground truth. We do this by giving the trained SpCycleGAN binary segmentation masks that indicate where to locate the nuclei. Since SpCycleGAN generates 2D slices, we sequentially generate 2D slices for each focal plane and stack them to construct a 3D synthetic volume with nuclei located where we told SpCycleGAN we wanted the nuclei. Hence, we have synthetic volumes and their corresponding ground truth. Thus SpCycleGAN is an unsupervised and unpaired method that requires no manually annotated images for training. Using this SpCycleGAN, we can create large numbers of synthetic ground truth masks and generate many corresponding synthetic microscopy volumes for training. In total, we generated 950 synthetic microscopy subvolumes ($128\times 128\times 128$ voxels) using representative subvolumes of different datasets (see Table 1).

**Figure 3:**
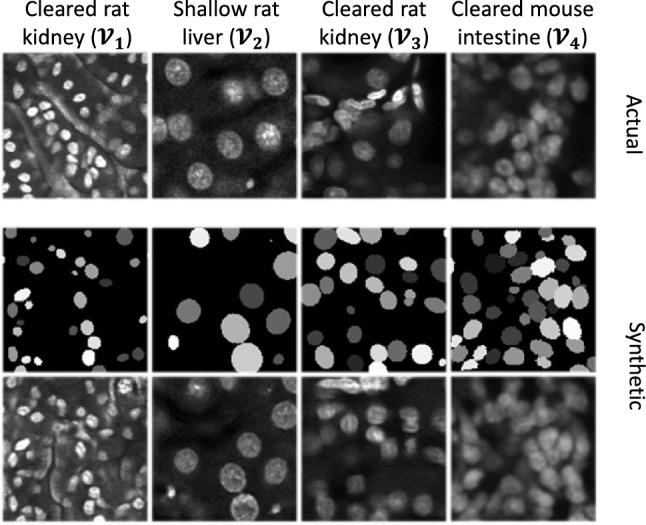
Generation of synthetic images and training data from exemplar datasets. Single optical *XY*-sections of actual microscopy images (top row), synthetic segmentation masks used by SpCycleGAN (middle row), and corresponding synthetic microscopy images (bottom row). ${\mathcal {V}}_1$ is rat kidney tissue that was fixed and cleared using the Scale^37^ technique, and then imaged by confocal microscopy. ${\mathcal {V}}_3$ is rat kidney tissue that was fixed, cleared using BABB^38^ and imaged using confocal microscopy. ${\mathcal {V}}_1$ differs from ${\mathcal {V}}_3$ in that ${\mathcal {V}}_1$ includes fluorescent objects that are not nuclei, and thus must be distinguished from nuclei.

Examples of actual microscopy images and corresponding synthetic microscopy images are shown in Fig. 3.

The synthetic images generated by SpCycleGAN were used to train the NISNet3D model. Further, to test the necessity of manually annotated images, we trained two versions of NISNet3D for two weighted models, NISNet3D-synth and NISNet3D-slim. NISNet3D-synth was trained using only synthetic datasets whereas NISNet3D-slim was trained on synthetic images and a small number of manually annotated actual microscopy volumes. The details of NISNet3D-synth and NISNet3D-slim training and evaluation are described in “Methods”.

### Evaluation of 3D segmentation—general approach

We compare NISNet3D with other deep learning image segmentation methods including VNet^24^, 3D U-Net^23^, Cellpose^3^, DeepSynth^6^, nnU-Net^45^ and StarDist3D^15^. In addition, we also compare NISNet3D to several non-deep learning segmentation methods including the 3D Watershed transform^46^, Squassh^47^, and the “IdentifyPrimaryObject” module from CellProfiler^48^. We used the above methods for comparison with NISNet3D because they have been commonly used in the literature^49–53^. We also use the 2D watershed transform and connected component analysis from VTEA for comparison^54^. We trained and evaluated the methods using the same dataset as used for the evaluation of NISNet3D. We also used the same training and evaluation strategies used for NISNet3D, as described in “Experimental settings”. It should be emphasized that this means we also use synthetic data to train the comparison methods similar to how we used synthetic data to train NISNet3D.

**Table 2:**
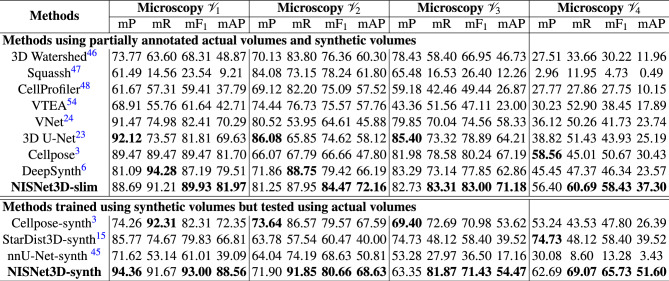
Comparison of object-based instance segmentation metrics for microscopy datasets in fluorescence datasets.

The best performance with respect to each metric is in bold. We use the mean Precision (mP), mean Recall (mR), mean F1 ($\mathrm {mF_1}$) score, and mean Average Precision (mAP) on multiple IoU thresholds. The methods above the double line are results using both synthetic and actual microscopy volumes for training, and use actual volumes for testing. The methods below the double line use only synthetic data for training but use actual volumes for testing.

**Table 3:** Comparison of object-based instance segmentation metrics in electron microscopy dataset NucMM.

Methods	Microscopy ${{\mathcal {V}}_5}$						
	mP	mR	${\textrm{mF}}_1$	${\textrm{AP}_{.50}}$	${\textrm{AP}_{.75}}$	mAP	AJI
StarDist3D^15^	73.44	74.24	73.84	88.59	9.78	61.20	68.56
DeepSynth^6^	81.63	76.04	78.72	71.47	50.88	63.74	75.54
Cellpose^3^	96.15	94.47	95.30	94.90	83.82	91.21	81.31
NISNet3D-slim	**96.89**	**96.24**	**96.56**	**95.98**	**88.84**	**93.62**	**83.90**

The best performance with respect to each metric are in bold. We use the mean Precision (mP), mean Recall (mR), mean F1 ($\mathrm {mF_1}$) score, and mean Average Precision (mAP) on multiple IoU thresholds. AJI is the Aggregated Jaccard Index^55^.

The parameters of all the comparison segmentation methods were manually adjusted to achieve the best visual segmentation results. We feel this is the best way for a fair comparison of the segmentation methods because we tried to allow each of the methods to demonstrate their best performance. This is further discussed in “Experimental settings”. The values of $\textrm{mP}$, $\textrm{mR}$, $\textrm{F}_1$, AP, and mAP were obtained for all of the methods mentioned above using the microscopy datasets ${\mathcal {V}}_1$–${\mathcal {V}}_5$ are shown in Tables 2 and 3. Note that in Table 2, the methods above the double line are results using both synthetic and partially annotated actual microscopy volumes for training. The methods below the double line use only synthetic data for training. We are surprised by how NISNet3D-synth, using only synthetic volumes for training, outperforms NISNet3D-slim that uses a combination of both synthetic data and partially annotated actual volumes in many cases. NISNet3D’s unique strength lies in instance segmentation, providing more accurate discrimination of individual nuclei. For qualitative evaluations the entire 3D volume was used, while for quantitative evaluations we use the annotated subvolumes. Therefore $\textrm{mP}$, $\textrm{mR}$, $\textrm{F}_1$, AP, and mAP are computed for each dataset.

### Qualitative and quantitative improvement of instance segmentation with NISNet3D

**Figure 4:**
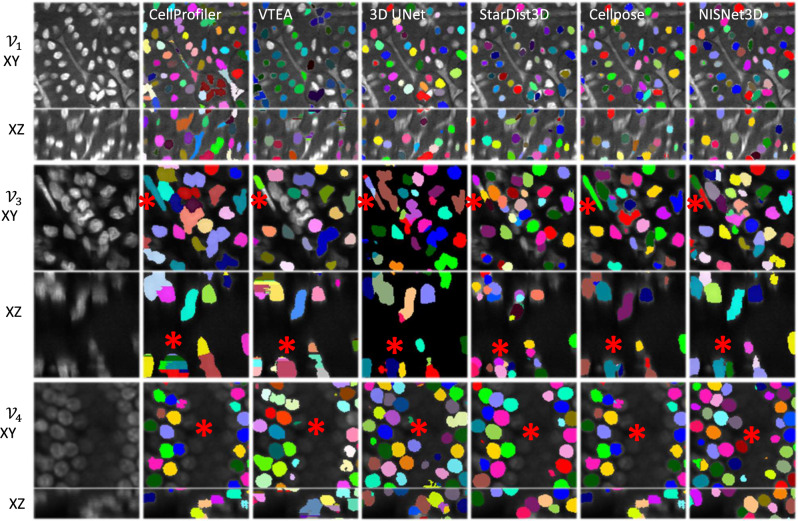
Qualitative assessment of NISNet3D segmentation in 2D and 3D. Three volumes were segmented by a 3D Cellprofiler “IdentifyPrimaryObject” module, 2D watershed and 3D connected components (VTEA), 3D U-Net, StarDist3D, Cellpose and NISNet3D. Shown are single optical section in *XY* or orthogonal sections of the image volume without (first column) or with unique segmented instances. NISNet3D demonstrates improved instance segmentation in *XY* and *XZ* imaging planes (red asterisks). We use different colors to distinguish different nuclei instances segmented by corresponding methods.

**Figure 5:**
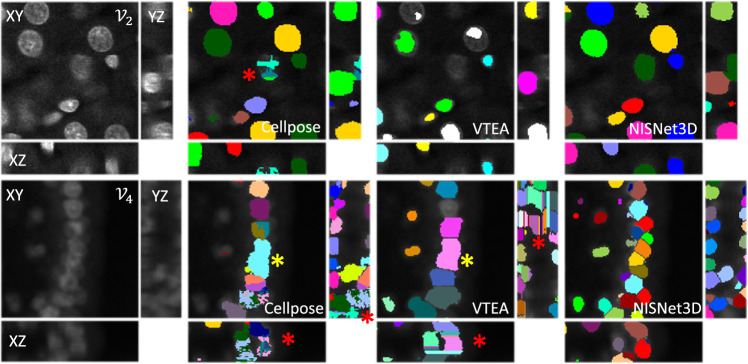
NISNet3D demonstrates improved instance segmentation. Two regions from (${\mathcal {V}}_2$ and ${\mathcal {V}}_4$) were segmented with 2D watershed transform and connected component analysis (VTEA), Cellpose, and NISNet3D. A single optical plane and *XZ* and *YZ* projections are shown with segmentation instance uniquely colored. NISNet3D improves semantic and instance segmentation over with examples of over- and under-segmentation (red vs yellow asterisks respectively) by both Cellpose or the 2D/3D connected component approach implemented in VTEA. Overall, NISNet3D provides the best instance segmentation in 3D.

Results presented in Figs. 4, 5, Supplementary Figures S4, and S5 show that, in general, the non-deep learning approaches (3D Watershed transform^46^, Squassh^47^, Cellprofiler’s “IdentifyPrimaryObject” module^48^, and VTEA’s 2D watershed transform and connected component analysis^54^) yielded heterogeneous segmentation results characterized by examples of both over- and under-segmentation. For example, VTEA’s 2D watershed transform and connected component analysis and Cellprofiler’s “IdentifyPrimaryObject” module are uniquely susceptible to axial over-segmentation (asterisks in *XZ* planes of ${\mathcal {V}}_3$ in Fig. 4), due to their dependence on fusing 2D segmentations into a final 3D volume. The deep learning approaches including StarDist3D^15^, Cellpose^3^, DeepSynth^6^ and nnU-Net^45^ performed far better in instance segmentation with some notable weaknesses compared to NISNet3D (Figs. 4, 5, Supplementary Figures S4, S5). For example, examination of the *XY* and *XZ* planes of ${\mathcal {V}}_3$ and ${\mathcal {V}}_4$ shown in Fig. 4 shows multiple examples of nuclei that were either over- or under-segmented by 3D U-Net, StarDist3D, and Cellpose, but were accurately detected and distinguished by NISNet (some indicated with asterisks). Results presented in Fig. 5 demonstrate that NISNet3D performs particularly well in the challenging setting of densely packed nuclei, particularly in poorly labeled samples. Asterisks indicate striking examples of nuclei that were accurately detected and distinguished by NISNet3D, but suffered from over- and under-segmentation by Cellpose and VTEA.

Overall, visual comparisons of segmentation results obtained from NISNet3D with those obtained from the Cellpose, DeepSynth, StarDist3D, 3D U-Net, and nnU-Net suggest the NISNet3D addresses segmentation weaknesses of these other deep learning techniques. To assess the qualitative improvement in segmentation results generated by NISNet3D, we measured quantitative metrics of instance and semantic segmentation on manually annotated image volumes. As the primary goal of nuclear segmentation is to detect nuclei, instance segmentation accuracy was evaluated using object-based metrics. To reduce bias^56^, we calculated the mean Precision, mean Recall and mean $\mathrm {F_1}$ scores with multiple IoU thresholds. We set $T_{\textrm{IoUs}}= \{0.25,0.3,\ldots ,0.45\}$ for datasets ${\mathcal {V}}_1$–${\mathcal {V}}_4$, and set $T_{\textrm{IoUs}}= \{0.5,0.55,\ldots ,0.75\}$ for dataset ${\mathcal {V}}_5$. Since segmenting 3D objects is more challenging than segmenting 2D objects, the IoU thresholds we used for evaluating 3D segmentation methods (0.25–0.5) are lower than the IoU thresholds reported for evaluating 2D segmentation methods (0.5–0.95), but are comparable to those reported in the literature for 3D segmentation^3,15,49,50,57–60^. The higher thresholds used for evaluation of nuclei in ${\mathcal {V}}_5$ reflect the fact that the nuclei in this volume are less densely packed, and thus overlap less. Note that these thresholds are not used by any of the segmentation methods and thus do not affect how the nuclei are segmented. For a given threshold, the common object detection metric Average Precision (AP)^61,62^ was calculated by computing the area under the Precision-Recall Curve^63^. In addition, we use the Aggregated Jaccard Index (AJI)^55^ to integrate instance and segmentation segmentation errors.

**Figure 6:**
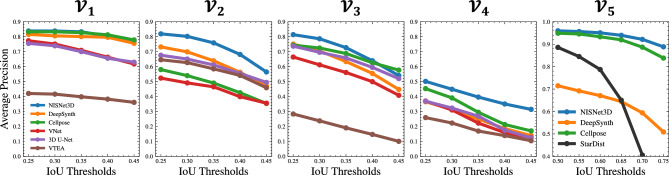
Evaluation results using average precision (AP) for multiple intersection-over-unions (IoUs) thresholds, $T_{\textrm{IoUs}}$, for datasets ${\mathcal {V}}_1$–${\mathcal {V}}_5$. Note we only show the methods that have higher performance.

NISNet3D had the highest $\textrm{mAP}$ and $\mathrm {mF_1}$ scores amongst the tested segmentation approaches. $\textrm{mP}$ and $\textrm{mR}$ were slightly lower in some cases without impacting the $\mathrm {mF_1}$ score. In these cases, where NISNet3D under-performed in $\textrm{mR}$ and $\textrm{mP}$, this is likely due to over and under segmentation errors from a large marker being generated. Importantly, when the IoU threshold is increased, NISNet3D continues to outperform the other tested approaches (Fig. 6). We observe that all approaches under-performed on ${\mathcal {V}}_4$ likely due to more crowded nuclei and under-sampling in the *Z*-axis. Analyses of the accuracy of semantic segmentation (shown in Supplementary Tables S1, S2) show that Cellpose^3^, StarDist^15^, nnU-Net^45^, VNet^24^, and 3D U-Net^23^ perform similarly with respect to distinguishing nuclei from background.

## Discussion

In this paper, we describe NISNet3D, a true 3D nuclei segmentation approach built on 3D U-Net^23^. Arguably, a significant bottleneck to the development and use of 3D deep learning approaches has been access to high quality training data^64^. Therefore, to facilitate training NISNet3D we developed a generative model based on our previous work on CycleGAN to make high quality training data without the need for ground truth^7,44^. To augment this generative approach, we also incorporated elastic deformations to generate non-ellipsoidal models of nuclei to better capture the diversity of nuclei found in tissues. Lastly, to address a need for better instance segmentation in 3D we also implemented an improved instance splitting approach.

We demonstrate that NISNet3D trained exclusively on our synthetic volumes (tested on actual microscopy volumes) performed as well as or better than NISNet3D trained on a combination of manually annotated and synthetic volumes. This suggests that synthetic volumes, made without any manually annotated volumes, are sufficient for training a 3D segmentation deep learning model to produce high-quality instance segmentations of cell nuclei. Further, although NISNet3D semantic segmentation accuracy was similar to other segmentation approaches tested here, NISNet3D instance segmentation was more accurate. We conclude that NISNet3D specifically improves instance segmentation of nuclei through a combination of better 3D training data and our instance-splitting approach.

We acknowledge that NISNet3D may have potential issues with segmenting nuclei that do not conform to our assumption that nuclei are only slightly deformed ellipsoids. For example, multi-lobed nuclei found in leukocytes or multi-nucleated myocytes are cases where our assumption may not hold. Morphological variability is a general issue in machine learning as training must involve all potential morphologies^6^. During the process of training, machine learning approaches derive features and characteristics from the contents of the training images. Thus, this weakness can be addressed by incorporating more models of varying nuclei morphology, ideally using our SpCyclGAN.

In the future, we will explore the generation of synthetic nuclei segmentation masks to better simulate complex nuclei morphologies and more realistic densities and distributions. Further, we are extending the current SpCycleGAN to generate fully 3D synthetic volumes to include a model of a microscope point spread function. This way, the generated synthetic volumes will more closely model the distribution of actual microscopy volumes, further improving synthetic volumes and the segmentation accuracy of approaches like NISNet3D.

## Methods

In this section, we provide more details on the structure of NISNet3D. In our experiments, we used both real annotated volumes and synthetic volumes to train the comparison methods similar to how we used synthetic data to train NISNet3D. We therefore provide more detail as to how we generated the synthetic training data.

### Notation and overview

In this paper, we denote a 3D image volume of size $X\times Y\times Z$ voxels by *I*, and a voxel having coordinates (*x*, *y*, *z*) in the volume by $I_{(x,y,z)}$. We use superscripts to distinguish between the different types of volumes and arrays. $I^{\textrm{orig}}$ will be used to denote an original microscopy volume. The objective is to segment nuclei within $I^{\textrm{orig}}$. The nuclei are labeled with unique voxel intensities and comprise the labeled volume $I^{\textrm{seg}}$. If we have ground truth from real microscopy volumes then the volume $I^{\textrm{label}}$ corresponds to the annotated nuclei with unique voxel intensities. This is done to distinguish each nuclei instance.

If we use synthetic images for training then $I^{\textrm{bi}}$ and $I^{\textrm{syn}}$ will be used to denote a generated synthetic binary segmentation masks and a synthetic microscopy volume, respectively. In addition, nuclei with corresponding masks in $I^{\textrm{bi}}$ are labeled with unique voxel intensities and to the volume $I^{\textrm{label}}$. This is done to distinguish each nuclei instance. In either case (actual or synthetic training data) $I^{\textrm{label}}$ will be used during the training of NISNet3D and comparison methods.

In addition to $I^{\textrm{label}}$, NISNet3D generates from $I^{\textrm{label}}$ a vector field array, $I^{\textrm{vec}}$ of size $X\times Y\times Z\times 3$, also for training purposes. Each element $I^{\textrm{vec}}_{(x,y,z)}$ of $I^{\textrm{vec}}$, located at (*x*, *y*, *z*), is a 3D vector $\mathbf {{\textbf {V}}}_{(x,y,z)}$ that is associated with the nucleus voxel $I^{\textrm{label}}_{(x,y,z)}$ and points to the centroid of the nucleus. Detailed description of vector field generation will be discussed in “Modified 3D U-Net”.

**Figure 7:**
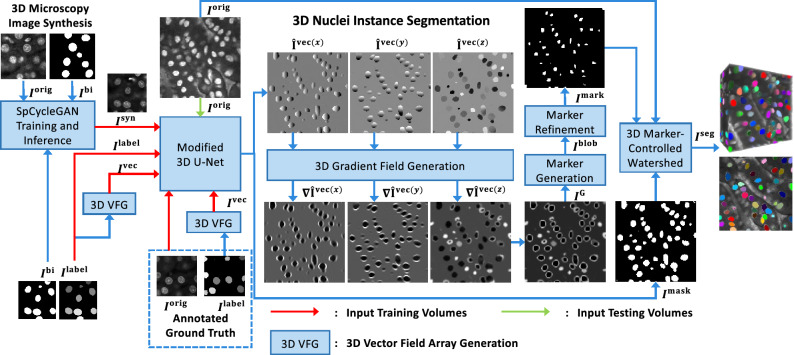
Overview of 3D nuclei instance segmentation using NISNet3D. The training and synthetic image generation are also shown but are not explicitly part of NISNet3D. NISNet3D is trained with synthetic microscopy volumes generated from SpCycleGAN and/or with annotated actual volumes as indicated. Note that $I^{\textrm{bi}}$ and $I^{\textrm{orig}}$ are not matched since they are part of the unpaired image-to-image translation of SpCyCleGAN.

For a given input volume, $I^{\textrm{orig}}$, NISNet3D generates a corresponding volume $I^{\textrm{mask}}$ of size $X\times Y\times Z\times 3$ comprising the binary segmentation results as well as an estimate of the vector field array, $\hat{I}^{\textrm{vec}}$, which is used to locate the nuclei centroids. In addition, $\hat{I}^{\textrm{vec}}$ is used to generate a 3D array of gradients, $I^{\textrm{G}}$ of size $X\times Y\times Z$, that is used in conjunction with morphological operations and watershed segmentation to distinguish and separate touching nuclei as well as remove small objects. The final output, $I^{\textrm{seg}}$, is a color-coded segmentation volume. An overview of the entire system is shown in Fig. 7.

### NISNet3D training and instance segmentation

In this section, we describe the NISNet3D architecture, how to train it, and then use NISNet3D for nuclei instance segmentation.

**Figure 8:**
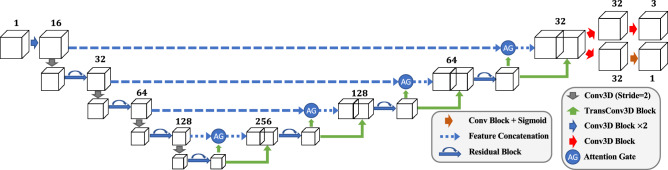
NISNet3D uses a modified 3D U-Net architecture with residual blocks, attention gates, and multi-task learning module.

#### Modified 3D U-Net

NISNet3D utilizes a modified 3D U-Net as shown in Fig. 8 which outputs the same size array as the input.

**Figure 9:**
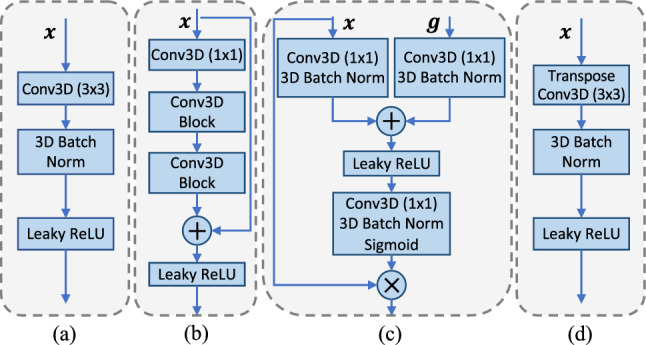
(**a**) Conv3D Block, (**b**) residual block, (**c**) attention gate, (**d**) TransConv3D block.

The 3D U-Net encoder consists of multiple Conv3D Blocks (Fig. 9a) and Residual Blocks (Fig. 9b)^65^. Instead of using max pooling layers, we use Conv3D Blocks with stride 2 for feature down-sampling, which introduces more learnable parameters. Each convolution block consists of a 3D convolution layer with filter size $3\times 3\times 3$, a 3D batch normalization layer, and a leaky ReLU layer. The decoder consists of multiple TransConv3D blocks (Fig. 9d) and attention gates (Fig. 9c). Each TransConv3D block includes a 3D transpose convolution with filter size $3\times 3\times 3$ followed by 3D batch normalization and leaky ReLU. We use a self-attention mechanism^66^ to refine the feature concatenation while reconstructing the spatial information.

##### Training the modified 3D U-Net

The modified 3D U-Net can be trained on both synthetic microscopy volumes $I^{\textrm{syn}}$ or actual microscopy volumes $I^{\textrm{orig}}$ if manual ground truth annotations are available.

As shown in Fig. 7, during training, NISNet3D’s modified 3D U-Net takes an $I^{\textrm{syn}}$ or $I^{\textrm{orig}}$, an $I^{\textrm{label}}$, and an $I^{\textrm{vec}}$ as input. $I^{\textrm{label}}$ is the $X\times Y\times Z$ gray-scale label volume corresponding to $I^{\textrm{bi}}$ where different nuclei are marked with unique pixel intensities. It used is used by NISNet3D to learn the segmentation masks. To facilitate segmentation NISNet3D identifies nuclei centroids and estimates their locations using a vector field, $I^{\textrm{vec}}$. In particular, $I^{\textrm{vec}}$ is part of the ground truth data used during training to enable the network to learn nuclei centroid locations. In addition, the 3D vector field array helps identify the boundaries of adjacent/touching nuclei since their corresponding vectors tend to point in different directions.

As indicated previously, a vector field, $I^{\textrm{vec}}$, is an $X\times Y\times Z\times 3$ array whose elements are 3D vectors that point to the centroids of the corresponding nuclei. Specifically, $I^{\textrm{vec}}_{(x,y,z)}$ is a 3D vector at location (*x*, *y*, *z*) and points to the nearest nucleus centroid. We also denote the $X\times Y\times Z$ array consisting of the first component of each 3D vector by $I^{\textrm{vec}(x)}$. Similarly, we denote the arrays of the second and third components by $I^{\textrm{vec}(y)}$ and $I^{\textrm{vec}(z)}$, respectively, that is $I^{\textrm{vec}}_{(x,y,z)} = (I^{\textrm{vec}(x)}_{(x,y,z)}, I^{\textrm{vec}(y)}_{(x,y,z)}, I^{\textrm{vec}(z)}_{(x,y,z)})$.

**Figure 10:**
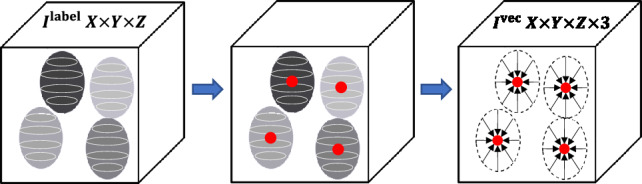
Steps for 3D vector field array generation (VFG). Each nucleus voxel in the 3D vector field array, $I^{\textrm{vec}}$ represents a 3D vector that points to the centroid of current nucleus.

The first step in the 3D vector field array generation (VFG) (see in Fig. 10) is to obtain the centroid of each nucleus in $I^{\textrm{label}}$. We denote the $k^{\textrm{th}}$ nucleus as the set of voxels with intensity *k* in $I^{\textrm{label}}$, and the location of the centroid of the $k^{\textrm{th}}$ nucleus by $(x_k, y_k, z_k)$. We define $\mathbf {{\textbf {V}}}^{k}_{(x,y,z)}$ to be the 3D vector from (*x*, *y*, *z*) to $(x_k, y_k, z_k)$ and set $I^{\textrm{vec}}_{(x,y,z)} = \mathbf {{\textbf {V}}}^{k}_{(x,y,z)}$ as long as the voxel at (*x*, *y*, *z*) is not a background voxel. Note that if a voxel $I^{\textrm{label}}_{(x,y,z)}$ is a background voxel (i.e. $I^{\textrm{label}}_{(x,y,z)}$=0), the corresponding entry $I^{\textrm{vec}}_{(x,y,z)}$ in the vector field array is set to **0**, as shown in Eq. (1).

Using $I^{\textrm{syn}}$ or $I^{\textrm{orig}}$, $I^{\textrm{label}}$, and $I^{\textrm{vec}}$, NISNet3D then outputs an estimate of the 3D vector field array $\hat{I}^{\textrm{vec}}$ ($\hat{I}^{\textrm{vec}(x)}$, $\hat{I}^{\textrm{vec}(y)}$, $\hat{I}^{\textrm{vec}(z)}$) and the 3D binary segmentation mask volume $I^{\textrm{mask}}$.

1 $$\begin{aligned} I^{\textrm{vec}}_{(x,y,z)}= {\left\{ \begin{array}{ll} \mathbf {{\textbf {V}}}^{k}_{(x,y,z)}, &{} \text {if } I^{\textrm{label}}_{(x,y,z)}\ne 0\\ {\textbf {0}},&{} \text {otherwise} \end{array}\right. } \end{aligned}$$

##### Loss functions

Given $I^{\textrm{syn}}$ or $I^{\textrm{orig}}$, $I^{\textrm{label}}$, and $I^{\textrm{vec}}$, the modified 3D U-Net simultaneously learns the nuclei segmentation masks $I^{\textrm{mask}}$ and the 3D vector field array $\hat{I}^{\textrm{vec}}$. In the modified 3D U-Net two branches but no sigmoid function are used to obtain $\hat{I}^{\textrm{vec}}$ because the vector represented at a voxel can point to anywhere in an array. In other words, the entries in $\hat{I}^{\textrm{vec}}$ can be negative numbers or large numbers. Unlike previous methods^28,67,68^ that directly learn the distance transform map, the 3D vector field array contains both the distance and direction of the nearest nuclei centroid from the current voxel location. This can help NISNet3D avoid the multiple detection of irregular shaped nuclei. The output 3D vector field array $\hat{I}^{\textrm{vec}}$ is compared with the ground truth vector field array $I^{\textrm{vec}}$ and the error between them minimized using the mean square error (MSE) loss function. Similarly, the segmentation result $I^{\textrm{mask}}$ is compared with the ground truth binary volume ${I}^{\textrm{bi}}$ and the difference minimized using a combination of the Focal Loss^69^ $\mathcal {L}_{\textrm{FL}}$ and Tversky Loss^70^ $\mathcal {L}_{\textrm{TL}}$ metrics. Denoting a ground truth binary volume ${I}^{\textrm{bi}}$ by *S*, the corresponding segmentation result $I^{\textrm{mask}}$ by $\hat{S}$, a ground truth vector field array $I^{\textrm{vec}}$ by *V* , and the estimated vector field array $\hat{I}^{\textrm{vec}}$ by $\hat{V}$, then, the entire loss function is,

2 $$\begin{aligned} \mathcal {L}(S,\hat{S},V,\hat{V}) = \lambda _3\mathcal {L}_{\textrm{TL}}(S,\hat{S}) +\lambda _4\mathcal {L}_{\textrm{FL}}(S,\hat{S}) + \lambda _5\mathcal {L}_{\textrm{MSE}}(V,\hat{V}) \end{aligned}$$

where

3 $$\begin{aligned}&\mathcal {L}_{\textrm{TL}} = \frac{\sum _{i=1}^{P}{s_{i1}\hat{s}_{i1}}}{\sum _{i=1}^{P}{s_{i1}\hat{s}_{i1}}+\alpha _1\sum _{i=1}^{P}{s_{i1}\hat{s}_{i0}}+\alpha _2\sum _{i=1}^{P}{s_{i0}\hat{s}_{i1}}},\nonumber \\&\mathcal {L}_{\textrm{FL}} = -\frac{1}{P}\sum _{i=1}^{P}{\{\beta s_{i1}\hat{s}_{i0}^{\gamma } \text {log}(\hat{s}_{i1})}+(1-\beta )s_{i0}\hat{s}_{i1}^{\gamma }\text {log}(\hat{s}_{i0})\},\nonumber \\&\mathcal {L}_{\textrm{MSE}} = \frac{1}{P}{\sum _{i=1}^{P}{(v_i-\hat{v}_i)^2}} \end{aligned}$$

where $\alpha _1 + \alpha _2=1$ are two hyper-parameters in Tversky loss^70^ that control the balance between false positive and false negative detections. $\beta$ and $\gamma$ are two hyper-parameters in Focal loss^69^ where $\beta$ balances the importance of positive/negative voxels, and $\gamma$ adjusts the weights for easily classified voxels. $v_i \in V$ is the $i^{\textrm{th}}$ element of *V*, and $\hat{v}_i \in \hat{V}$ is the $i^{\textrm{th}}$ entry in $\hat{V}$. Similarly, $s_i \in S$ is the $i^{\textrm{th}}$ voxel in *S*, and $\hat{s}_i \in \hat{S}$ is the $i^{\textrm{th}}$ voxel in $\hat{S}$. We define $\hat{s}_{i0}$ to be the probability that the $i^{\textrm{th}}$ voxel in $\hat{S}$ is a nuclei, and $\hat{s}_{i1}$ as the probability that the $i^{\textrm{th}}$ voxel in $\hat{S}$ is a background voxel. Similarly, $s_{i1}=1$ if $s_i$ is a nuclei voxel and 0 if $s_i$ is a background voxel, and vice versa for $s_{i0}$. Lastly, *P* is the total number of voxels in a volume.

##### Divide and conquer

**Figure 11:**
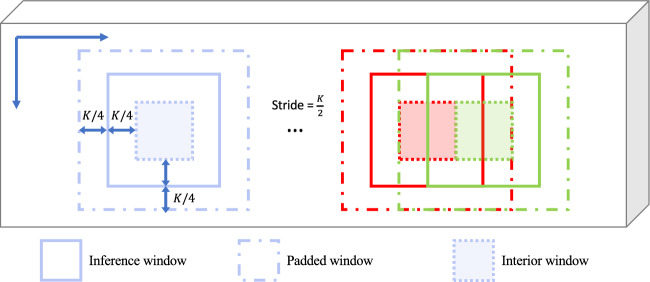
Proposed divide-and-conquer scheme for segmenting large microscopy volumes.

To segment a large microscopy volume, we propose a divide-and-conquer scheme shown in Fig. 11. We use an analysis window of size $K\times K\times K$ that slides along each original microscopy volume $I^{\textrm{orig}}$ of size $X\times Y\times Z$ and crops a subvolume. Considering that cropping may result in some nuclei being partially included, and that these partially included nuclei which lie on the border of the analysis window may cause inaccurate segmentation results, we construct a padded window by symmetrically padding each cropped subvolume by $\frac{K}{4}$ voxels on each border. Also, the stride of the moving window is set to $\frac{K}{2}$ so with every step it slides, it will have $\frac{K}{2}$ voxels overlapping with the previous window. For the segmentation results of every $K\times K\times K$ window, only the interior and centered $\frac{K}{2}\times \frac{K}{2}\times \frac{K}{2}$ subvolume, which we denoted as the interior window, will be used as the segmentation results. In this paper, we use $K=128$ for all testing data. Once the analysis window slides along the entire volume, a segmentation volume $I^{\textrm{mask}}$ and 3D vector field array $\hat{I}^{\textrm{vec}}$ of size $X\times Y\times Z$ will be generated. In this way, we can analyze any size input volume, especially very large volumes.

#### 3D nuclei instance segmentation

Based on the output of the modified 3D U-Net, a 3D gradient field and an array of markers are generated and used to separate densely clustered nuclei. Moreover, the markers are refined to attain better separation. As mentioned previously, each element/vector in the estimated 3D vector field array $\hat{I}^{\textrm{vec}}$ point to the centroid of the nearest nucleus. Most often, the vectors associated with voxels on the boundary of two touching nuclei point in different directions that correspond to the locations of the centroids of the touching nuclei, and hence have a large difference/gradient. We employ such larger gradients to detect the boundaries of touching and also overlapping nuclei.

Define $\nabla \hat{I}^{\textrm{vec}}$ as the gradient of $\hat{I}^{\textrm{vec}}$ which is obtained as given as:

4 $$\begin{aligned} \nabla \hat{I}^{\textrm{vec}}&= \begin{bmatrix} \nabla \hat{I}^{\textrm{vec}(x)}&\nabla \hat{I}^{\textrm{vec}(y)}&\nabla \hat{I}^{\textrm{vec}(z)} \end{bmatrix}^T\nonumber \\&=\begin{bmatrix} \frac{\partial \hat{I}^{\textrm{vec}(x)}}{\partial x}&{}\frac{\partial \hat{I}^{\textrm{vec}(y)}}{\partial y}&{}\frac{\partial \hat{I}^{\textrm{vec}(z)}}{\partial z}\\ \end{bmatrix}^T\nonumber \\&=\begin{bmatrix} S_x*\hat{I}^{\textrm{vec}(x)}&S_y*\hat{I}^{\textrm{vec}(y)}&S_z*\hat{I}^{\textrm{vec}(z)} \end{bmatrix}^T, \end{aligned}$$

where $\hat{I}^{\textrm{vec}(x)}$, $\hat{I}^{\textrm{vec}(y)}$, and $\hat{I}^{\textrm{vec}(z)}$ are the *x*, *y*, and *z* sub-arrays of the estimated 3D vector field array $\hat{I}^{\textrm{vec}}$ respectively, $S_x$, $S_y$, $S_z$ are 3D Sobel filters, and $*$ is the convolution operator. We also define the gradient map $I^{\textrm{G}}$ as the maximum of the gradient components along the *x*, *y* and *z* directions :

5 $$\begin{aligned} I^{\textrm{G}}&= \text {max}(\nabla \hat{I}^{\textrm{vec}(x)}, \nabla \hat{I}^{\textrm{vec}(y)}, \nabla \hat{I}^{\textrm{vec}(z)}) \end{aligned}$$

Elements of $I^{\textrm{G}}$ that correspond to boundaries of touching nuclei have larger values (larger gradients) which can be used to identify individual nuclei. We subsequently threshold $I^{\textrm{G}}$ via a thresholding function $\tau (x,T_{\textrm{m}})$ where $\tau (x,T_{\textrm{m}})=1$ if $x\ge T_{\textrm{m}}$ otherwise 0. This step is used to highlight and distinguish boundary voxles of individual nuclei from spurious edges that may arise from finding the gradients. The value of $T_{\text{m}}$ can affect the number of voxels delineated as boundary voxels. Larger values of $T_{\textrm{m}}$ result in thinner boundaries whereas smaller values of $T_{\textrm{m}}$ result in a thicker nuclei boundaries. We then subtract the result from the binary segmentation mask $I^{\textrm{mask}}$ obtained from the modified 3D U-Net, and then apply that to a non-linear half wave rectifier/RELU activation function $\sigma (\cdot )$ that sets all negative values to 0. We denote the result as $I^{\textrm{blob}}$ as given in Eq. (6). The difference $I^{\textrm{mask}}-\tau (I^{\textrm{G}}, T_{\textrm{m}})$ removes boundary voxles of touching nuclei while emphasizing the interior. Since the difference can be negative in some cases, we use $\sigma (\cdot )$ to eliminate negative values. Hence, $I^{\textrm{blob}}$ highlights the interior regions of nuclei which maybe used as markers to perform watershed segmentation^46,71^.

6 $$\begin{aligned} I^{\textrm{blob}}&= \sigma (I^{\textrm{mask}}-\tau (I^{\textrm{G}}, T_{\textrm{m}})) \end{aligned}$$

7 $$\begin{aligned} I^{\textrm{mark}}&= \delta _{t_{\textrm{f}}}(\delta _{t_{\textrm{c}}}(I^{\textrm{blob}},B_{\textrm{c}}),B_{\textrm{f}}) \end{aligned}$$

**Figure 12:**
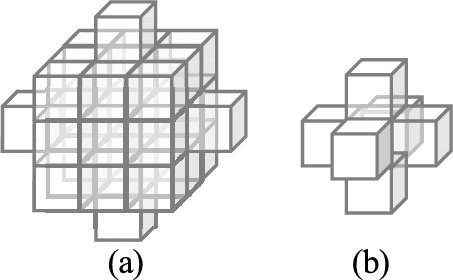
(**a**) coarse 3D structuring element $B_{\text{c}}$ and (**b**) fine 3D structuring element $B_{\text{f}}$ for 3D conditional erosion.

However, in some cases $I^{\textrm{blob}}$ may still contain boundary information. To refine it we use 3D conditional erosion with a coarse structuring element $B_{\textrm{c}}$ and a fine structuring $B_{\textrm{f}}$ as shown in Fig. 12. To achieve this we first identify the connected components^72^ in $I^{\textrm{blob}}$ and then iteratively erode each connected component using $B_{\textrm{c}}$ until its size is smaller than some threshold $t_{\textrm{c}}$. We then continue eroding each connected component using $B_{\textrm{f}}$ until its size is smaller than another threshold $t_{\textrm{f}}$. We denote the final result by $I^{\textrm{mark}}$, as shown in Eq. (7), where $\delta _{t_{\textrm{c}}}(I^{\textrm{blob}},B_{\textrm{c}})$ defines iterative erosion of all connected component in $I^{\textrm{blob}}$ by a coarse structuring element $B_{\textrm{c}}$ until the size of each component is smaller than the coarse object threshold $t_{\textrm{c}}$. Similarly, $\delta _{t_{\textrm{f}}}(I^{\textrm{blob}},B_{\textrm{f}})$, denotes iterative erosion by a fine structuring element, $B_{\textrm{f}}$, until the size of the object is smaller than the fine object threshold $t_{\textrm{f}}$. Finally, marker-controlled watershed^46^ is used to generate instance segmentation masks $I^{\textrm{seg}}$ using $I^{\textrm{mark}}$. Small objects in $I^{\textrm{seg}}$ that are less than 20 voxels in size are then removed, and each object color coded for visualization.

### 3D nuclei image synthesis

Deep learning methods generally require large amounts of training samples to achieve accurate results. However, manually annotating ground truth is a tedious task and impractical in many situations especially for large 3D microscopy volumes. NISNet3D, like any other trainable segmentation method, can use synthetic 3D volumes, annotated actual 3D volumes, or combinations of both as demonstrated here.

To generate synthetic volumes, we first generate synthetic segmentation masks that are used as ground truth masks, and then translate the synthetic segmentation masks into synthetic microscopy volumes using an unsupervised image-to-image translation model known as SpCycleGAN^7^.

The way SpCycleGAN works is that we first train SpCycleGAN and then after it is trained, we can generate 3D volumes with its own inherent ground truth. We do this by giving the trained SpCycleGAN binary segmentation masks that indicate where we want the nuclei to be located in synthetic image. SpCycleGAN will then generate a synthetic volume with nuclei located where we told SpCycleGAN we wanted the nuclei. Hence, we have synthetic volumes and their corresponding ground truth. It should be noted that we do not need any ground truth volumes to train SpCycleGAN.

#### Synthetic segmentation mask generation

To generate a volume of synthetic binary nuclei segmentation masks, we iteratively add *N* initial binary nuclei to an empty 3D volume of size $128\times 128\times 128$. Each initial nuclei is modeled as a 3D binary ellipsoid having random size, orientation, and location. The size, orientation, and location of the $n^{\textrm{th}}$ ellipsoid are parameterized by $\varvec{a}^{(n)}$, $\varvec{\theta }^{(n)}$, and $\varvec{t}^{(n)}$, respectively, where $\varvec{a}^{(n)}=[a_x^{(n)}, a_y^{(n)}, a_z^{(n)}]^T$ denotes a vector of semi-axes lengths of the $n^{\textrm{th}}$ ellipsoid, $\varvec{\theta }^{(n)}=[\theta _x^{(n)}, \theta _y^{(n)}, \theta _z^{(n)}]^T$ denotes a vector of rotation angles relative to the *X*, *Y*, and *Z* coordinate axes, and $\varvec{t}^{(n)}=[t_x^{(n)}, t_y^{(n)}, t_z^{(n)}]^T$ denotes the displacement/location vector of the $n^{\textrm{th}}$ ellipsoid relative the origin. The parameters $\varvec{a}^{(n)}$, $\varvec{\theta }^{(n)}$, and $\varvec{t}^{(n)}$ are randomly selected based on observations of nuclei characteristics in actual microscopy volumes.

Let $J^{\textrm{nuc},(n)}_{(x, y, z)}$ denote the $n^{\textrm{th}}$ initial nucleus at location (*x*, *y*, *z*) having intensity/label $n\in \{1,\ldots ,N\}$, then

8 $$\begin{aligned} J^{\textrm{nuc},(n)}_{(x, y, z)} = {\left\{ \begin{array}{ll} n, &{} \text {if } (\frac{x}{a_x^{(n)}})^2+(\frac{y}{a_y^{(n)}})^2+(\frac{z}{a_z^{(n)}})^2<1 \\ 0,&{} \text {otherwise} \end{array}\right. } \end{aligned}$$

The ellipsoids are assigned different intensity values to differentiate them from each other. Subsequently, each $J^{\textrm{nuc},(n)}_{(x, y, z)}$ undergoes a random translation given by $\varvec{t}^{(n)}$ followed by random rotations specified in $\varvec{\theta }^{(n)}$. Denoting the original coordinates by ${\textbf {X}}$ and the translated and rotated coordinates by $\tilde{{\textbf {X}}}$, respectively then

9 $$\begin{aligned} \tilde{{\textbf {X}}}=\begin{bmatrix} \tilde{x}\\ \tilde{y}\\ \tilde{z} \end{bmatrix}=R_z(\theta _z^{(n)})R_y(\theta _y^{(n)})R_x(\theta _x^{(n)}) \begin{bmatrix} x+t_x^{(n)}\\ y+t_y^{(n)}\\ z+t_z^{(n)} \end{bmatrix} \end{aligned}$$

where $R_x(\theta _x^{(n)})$, $R_y(\theta _y^{(n)})$, and $R_z(\theta _z^{(n)})$ denote the rotation matrices relative to the *X*, *Y*, and *Z* axes by angles $\theta _x^{(n)}$, $\theta _y^{(n)}$, and $\theta _z^{(n)}$, respectively. Thus, the final $n^{\textrm{th}}$ ellipsoid $I^{\textrm{nuc},(n)}_{(x, y, z)}$ is given by $I^{\textrm{nuc},(n)}_{(x, y, z)} = J^{\textrm{nuc},(n)}_{(\tilde{x}, \tilde{y}, \tilde{z})}$ for all $n\in \{1,\ldots ,N\}$. We finally constrain the transformed nuclei such that they do not overlap by more than $t_{\textrm{ov}}$ voxels. The ellipsoids $I^{\textrm{nuc},(n)}_{(x, y, z)}$, $n\in \{1,\ldots ,N\}$, are then used to fill in a binary volume $I^{\textrm{bi}}$ of size $X\times Y \times Z$.

Since actual nuclei are not strictly ellipsoidal but look more like deformed ellipsoids (see Fig. 2 (left column)), we use an elastic transformation^40,73^ to deform $I^{\textrm{bi}}$. To achieve this we first generate a coarse displacement vector field, $I^{\textrm{coarse}}$, which is an array of size $d\times d\times d\times 3$, whose entries are independent and Gaussian distributed random variables having zero mean and variance $\sigma ^2$ (that is $I_{(k,x,y,z)}^{\textrm{coarse}}\sim \mathcal {N}(0, \sigma ^2)$). The size parameter *d* is used to control the amount of deformation applied to the nuclei. $I^{\textrm{coarse}}$ is then interpolated to size $X\times Y\times Z\times 3$, via spline interpolation^74^ or bilinear interpolation^40^ to produce a smooth displacement vector field $I^{\textrm{smooth}}$. The entry $I^{\textrm{smooth}}_{(x,y,z,1)}$ indicates the distance by which voxel $I^{\textrm{bi}}_{(x,y,z)}$ will be shifted along the *X*-axis. Similarly, the elements $I^{\textrm{smooth}}_{(x,y,z,2)}$ and $I^{\textrm{smooth}}_{(x,y,z,3)}$ provide the distances by which $I^{\textrm{bi}}_{(x,y,z)}$ will be shifted along the *Y* and *Z*-axes, respectively. In our experiments, we used spline interpolation and values for $d \in \{4,5,10\}$. Each element in $I^{\textrm{coarse}}$ serves as an anchor point controlling the magnitude of elastic transform. Larger values of *d* result in larger deformations being applied through $I^{\textrm{smooth}}$ whereas smaller *d* values produce less deformation. Examples of deformed ellipsoids are shown in Fig. 2 (right column).

#### Synthetic microscopy volume generation

In the previous section, we described how we generate binary segmentation masks. Here we describe how we use the masks to generate synthetic microscopy volumes that have nuclei located as determined the binary segmentation mask. In particular, we use the unpaired image-to-image translation model known as SpCycleGAN^7^ for generating synthetic microscopy volumes. SpCycleGAN^7^ is a variation of CycleGAN^44^ where an additional spatial constraint is added to the loss function to maintain the spatial locations of the nuclei.

**Figure 13:**
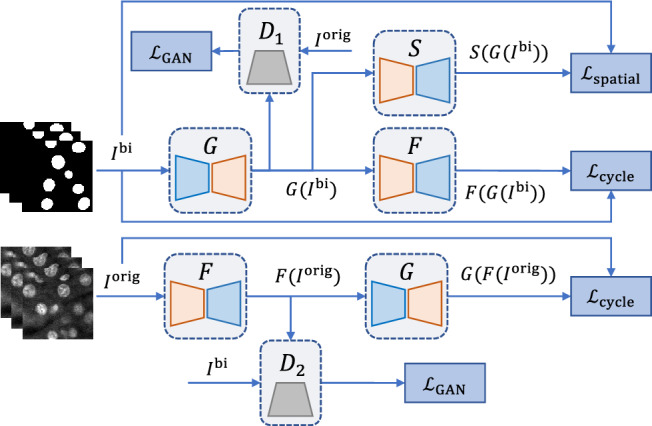
The architecture of SpCycleGAN that is used for generating synthetic microscopy volumes.

SpCycleGAN is shown in Fig. 13. The way SpCycleGAN works is that we first train SpCycleGAN and then after it is trained, we can generate 3D volumes with known ground truth. We do this by giving the trained SpCycleGAN binary segmentation masks that indicate where we want the nuclei to be located. SpCycleGAN will then generate a synthetic volume with nuclei located where we told SpCycleGAN we wanted the nuclei. Hence, we have synthetic volumes and their corresponding ground truth. In fact, the synthetic 3D volumes we generate using SpCycleGAN do not need any ground truth annotations at all because SpCycleGAN is an unsupervised and unpaired method. We can create many synthetic ground truth masks and generate many corresponding synthetic microscopy volumes that have nuclei located at the locations we choose using SpCycleGAN. By unpaired we mean that the inputs to SpCycleGAN are volumes of binary segmentation masks and actual microscopy images that do not correspond with each other. In addition, the binary segmentation masks created above are not used as ground truth volumes for performing segmentation of actual microscopy images.

As shown in Fig. 7, we use slices from volumes of the binary segmentation masks, $I^{\textrm{bi},(m)}, m\in \{1,\ldots ,M\}$, where *M* is the number of training samples/volumes, and slices from actual microscopy volumes $I^{\textrm{orig},(m)}, m\in \{1,\ldots ,M\}$ for training SpCycleGAN. After training we generate *L* synthetic microscopy volumes, $I^{\textrm{syn},(l)}, l\in \{1,\ldots ,L\}$, using synthetic microscopy segmentation masks, $I^{\textrm{bi},(l)}, l\in \{1,\ldots ,L\}$ other than the ones used for training. Note that since SpCycleGAN generates 2D slices, we use the slices to construct a 3D synthetic volume. We acknowledge that this can be a problem because SpCycleGAN may not capture information across the volume stack.

SpCycleGAN consists of two generators *G* and *F*, two discriminators $D_1$ and $D_2$. *G* learns the mapping from $I^{\textrm{bi}}$ to $I^{\textrm{orig}}$ whereas *F* performs the reverse mapping. Also, SpCycleGAN introduced a segmentor *S* for maintaining the spatial location between $I^{\textrm{bi}}$ and $F(G(I^{\textrm{bi}}))$. The entire loss function of SpCycleGAN is:

10 $$\begin{aligned} \mathcal {L}(G, F, S, D_1,D_2)&=\mathcal {L}_{\textrm{GAN}}(G,D_1, I^{\textrm{bi}},I^{\textrm{orig}})\nonumber \\&\quad +\mathcal {L}_{\textrm{GAN}}(F,D_2,I^{\textrm{orig}}, I^{\textrm{bi}})\nonumber \\&\quad +\lambda _1 \mathcal {L}_{\textrm{cycle}}(G,F,I^{\textrm{orig}}, I^{\textrm{bi}}) \nonumber \\&\quad + \lambda _2 \mathcal {L}_{\textrm{spatial}}(G,S,I^{\textrm{orig}}, I^{\textrm{bi}}) \end{aligned}$$

where $\lambda _1$ and $\lambda _2$ are weight coefficients controlling the loss balance between $\mathcal {L}_{\textrm{cycle}}$ and $\mathcal {L}_{\textrm{spatial}}$, and

11 $$\begin{aligned} \nonumber \mathcal {L}_{\textrm{GAN}}(G,D_1, I^{\textrm{bi}},I^{\textrm{orig}})&={{\,\mathrm{\mathbb {E}}\,}}_{I^{\textrm{orig}}}[\text {log}(D_1(I^{\textrm{orig}}))]\\ \nonumber&\quad +{{\,\mathrm{\mathbb {E}}\,}}_{I^{\textrm{bi}}}[\text {log}(1-D_1(G(I^{\textrm{bi}})))]\\ \nonumber \mathcal {L}_{\textrm{GAN}}(F,D_2, I^{\textrm{orig}},I^{\textrm{bi}})&={{\,\mathrm{\mathbb {E}}\,}}_{I^{\textrm{bi}}}[\text {log}(D_2(I^{\textrm{bi}}))]\\ \nonumber&\quad +{{\,\mathrm{\mathbb {E}}\,}}_{I^{\textrm{orig}}}[\text {log}(1-D_2(F(I^{\textrm{orig}})))]\\ \nonumber \mathcal {L}_{\textrm{cycle}}(G,F,I^{\textrm{orig}}, I^{\textrm{bi}})&={{\,\mathrm{\mathbb {E}}\,}}_{I^{\textrm{bi}}}[\Vert F(G(I^{\textrm{bi}}))-I^{\textrm{bi}}\Vert _1]\\ \nonumber&\quad +{{\,\mathrm{\mathbb {E}}\,}}_{I^{\textrm{orig}}}[\Vert G(F(I^{\textrm{orig}}))-I^{\textrm{orig}}\Vert _1]\\ \mathcal {L}_{\textrm{spatial}}(G, S, I^{\textrm{bi}}, I^{\textrm{orig}})&={{\,\mathrm{\mathbb {E}}\,}}_{I^{\textrm{bi}}}[\Vert S(G(I^{\textrm{bi}}))-I^{\textrm{bi}}\Vert _2] \end{aligned}$$

where $\Vert \cdot \Vert _1$ and $\Vert \cdot \Vert _2$ denote the $L_1$ norm and $L_2$ norm, respectively. ${{\,\mathrm{\mathbb {E}}\,}}_{I}$ is the expected value over all the input volumes of a batch to the network.

We use 512 slices of ${\mathcal {V}}_1$, 512 slices of ${\mathcal {V}}_2$, 4096 slices of ${\mathcal {V}}_3$, and 2048 slices of ${\mathcal {V}}_4$ for training SpCycleGAN.

There is a limitation of our method since we need to model the nuclei shape in the synthetic binary mask as close as possible to the real nuclei in the microscopy volume. This way SpCycleGAN can generate more realistic microscopy images.

### Comparison methods

We compared NISNet3D with several deep learning image segmentation methods including VNet^24^, 3D U-Net^23^, Cellpose^3^, DeepSynth^6^, nnU-Net^45^ and StarDist3D^15^. In addition, we also compare NISNet3D with several commonly used biomedical image analysis tools and software including 3D Watershed^46^, Squassh^47^, and Cellprofiler’s “IdentifyPrimaryObject” module^48^. We also compare to a blob-slice method using the 2D watershed transform with 3D connected components from VTEA^54^. In the cases of software packages Cellprofiler and VTEA, we configured them for segmentation using our data sets. The details are described in the next subsection.

VNet^24^ and 3D U-Net^23^ are two popular 3D encoder-decoder networks with shortcut concatenations designed for biomedical image segmentation. Cellpose^3^ uses a modified 2D U-Net for estimating image segmentation and spatial flows, and uses a dynamic system^3^ to cluster pixels and further separate touching nuclei. When segmenting 3D volumes, Cellpose works from three different directions slice by slice and combines the 2D segmentation results into a 3D segmentation volume^3^. DeepSynth uses a modified 3D U-Net to segment 3D microscopy volumes and the watershed transform to separate touching nuclei^6^. nnU-Net^45^ is a self-configuring U-Net-based method that mainly does semantic segmentation for biomedical applications and uses connected component analysis of the center class, followed by iterative outgrowing into the nuclei border region on the semantic segmentation results. Similarly, StarDist3D uses a modified 3D U-Net to estimate the star-convex polyhedra used to represent nuclei^15^.

Alternative non-deep learning based techniques such as 3D Watershed^46^ uses the watershed transformation^71^ and conditional erosion^46^ for nuclei segmentation. Similarly, Squassh, an ImageJ plugin for both 2D and 3D microscopy image segmentation, uses active contours^47^. CellProfiler is an image processing toolbox and provides customized image processing and analysis modules^48^ We used Cellprofiler’s “IdentifyPrimaryObject” module for our study. VTEA is an ImageJ plugin that combines various approaches including Otsu’s thresholding and the watershed transform to segment 2D nuclei slice by slice and reconstruct the results into a 3D volume^54^.

For comparison purposes, we trained and evaluated the methods above using the same dataset as used for NISNet3D. We also used the same training and evaluation strategies used for NISNet3D as described in “Experimental settings”. This means that the methods that required training were also trained on synthetic data. We provide a discussion of the results in “Discussion”. Note that the 3D Watershed transform, Squassh, CellProfiler, and VTEA do not need to be trained because they use more traditional image analysis techniques.

nnU-Net is primarily used for semantic segmentation for biomedical applications^36,45^. In order to obtain nuclei instance segmentation, nnU-Net uses post-processing with connected component analysis of the center class, followed by iterative outgrowing into the nuclei border region from the semantic segmentation results. We used nnU-Net’s post-processing steps to obtain the instance segmentation results presented. We observe that nnU-Net has difficulty separately touching nuclei on an object-level basis. This is due to the connected component analysis during the post-processing where touching nuclei will be grouped as one object.

### Experimental settings

The parameters used for generating $I^{\textrm{bi}}$ are provided in Table 4 where $(a_{\textrm{min}}$, $a_{\textrm{max}})$ is the range of the ellipsoid semi-axes lengths, $t_{\textrm{ov}}$ is the maximum allowed overlapping voxels between two nuclei, and *N* is the total number of nuclei in a synthetic volume. These parameters are based on visual inspection of nuclei characteristics in actual microscopy volumes.

**Table 4:** The training evaluation strategies for NISNet3D and comparison methods.

Parameters for generating synthetic microscopy volumes							NISNet3D-slim training strategies		NISNet3D-slim
Volume name	$\varvec{a_{\textrm{min}}}$	$\varvec{a_{\textrm{max}}}$	$\varvec{t_{\textrm{o}}}$	$\varvec{N}$	$\varvec{d}$	$\varvec{\sigma }$	Version	Training	Evaluation
${\mathcal {V}}_1$	4	8	5	500	10	1	$\mathcal {M}_1$	50 volumes of synthetic ${\mathcal {V}}_1$	Tested on 1 subvolume of ${\mathcal {V}}_1$
${\mathcal {V}}_2$	10	14	10	70	4	1	$\mathcal {M}_2$	50 volumes of synthetic ${\mathcal {V}}_2$	Tested on 16 subvolumes of ${\mathcal {V}}_2$
${\mathcal {V}}_3$	6	10	200	200	5	2	$\mathcal {M}_3$	50 volumes of synthetic ${\mathcal {V}}_3$	3-fold cross-validated on 9 subvolumes of ${\mathcal {V}}_3$
${\mathcal {V}}_4$	8	10	100	560	4	4	$\mathcal {M}_4$	lightly retrained using $\mathcal {M}_3$ on 1 subvolume of ${\mathcal {V}}_4$	Tested on 4 subvolumes of ${\mathcal {V}}_4$
${\mathcal {V}}_5$	–	–	–	–	–	–	$\mathcal {M}_5$	27 subvolumes of ${\mathcal {V}}_5$	Tested on 27 subvolumes of ${\mathcal {V}}_5$

SpCycleGAN was trained on unpaired $I^{\textrm{bi}}$ and $I^{\textrm{orig}}$ and the trained model was used to generate synthetic microscopy volumes. The weight coefficients of the loss functions were set to $\lambda _1 = \lambda _2 = 10$ for the SpCycleGAN^7^. The synthetic microscopy volumes were verified by a biologist (co-author Dunn).

For the 3D Watershed transform, we used a 3D Gaussian filter to preprocess the images and used Otsu’s method to segment/isolate objects from the background of each volume. Subsequently the 3D conditional erosion described in “3D nuclei instance segmentation” was used to obtain the markers, and the marker-controlled 3D watershed method, which was implemented by the Python Scikit-image library, was used to separate touching nuclei.

For Cellprofiler, we used customized image processing modules including inhomogeneity correction, median filtering, and morphological erosion were used to preprocess the images, and the default “IdentifyPrimaryObject” module was used to obtain 2D segmentation masks for each slice. “IdentifyPrimaryObjects” uses the 2D watershed transform segmentation for identification of objects in 3D biological structures^48^.

With regards to Squassh^47^, we used “Background subtraction” with a manually tuned “rolling ball window size” parameter. The rest of the parameters were set to their default values. We observed that Squassh did fairly well on volume ${\mathcal {V}}_2$ but totally failed on ${\mathcal {V}}_4$ due to the densely clustered nuclei. For VTEA^54^, a Gaussian filter and background subtraction were used to preprocess the image. The object building method in VTEA was set to “Connect 3D”, and the segmentation threshold determined automatically. We manually tuned the parameters “Centroid offset”, “Min vol”, and “Max vol” to obtain the best visual segmentation results. Finally, the watershed transform was used for 2D segmentation. The 2D segmentations were then merged into a 3D segmentation volume using a blob-slice method^54,75^. For Cellpose, we used the “nuclei” style and since the training of Cellpose is only limited to 2D images, we trained Cellpose on every *XY* focal planes of our subvolumes following the training schemes given in Table 4. For nnU-Net, we trained nnU-Net for 500 epochs using the “3d_fullres” configuration.

For VNet^24^, 3D U-Net^23^, and DeepSynth^6^ we improved the segmentation results by using our 3D conditional erosion described in “3D nuclei instance segmentation” followed by 3D marker-controlled watershed transform to separate touching nuclei.

Both SpCycleGAN and NISNet3D were implemented using PyTorch. We used 9-block ResNet for the generators *G*, *F*, and the segmentor *S* (see Fig. 13). The discriminators $D_1$ and $D_2$ (Fig. 13) were implemented with the “PatchGAN” classifier^76^. SpCycleGAN was also trained with an Adam optimizer^77^ for 200 epochs with an initial learning rate 0.0002 that linearly decays to 0 after the first 100 epochs. Figure 2.3 depicts the generated synthetic nuclei segmentation masks and corresponding synthetic microscopy images.

As mentioned above, we investigated two versions of NISNet3D: NISNet3D-slim (which includes 5 models $\mathcal {M}_1$–$\mathcal {M}_5$) and NISNet3D-synth (which includes 1 model $\mathcal {M}_{\textrm{syn}}$) for different application scenarios. NISNet3D-synth is designed for the situation where no ground truth annotations are available. In contrast, NISNet3D-slim is used for the case where limited ground truth annotated volumes are available. In the case of NISNet3D-slim, synthetic volumes are used for training and a small amount of actual ground truth data is for used for light retraining. We train NISNet3D using both of the strategies to show the effectiveness of our approach. We trained 5 versions of NISNet3D-slim, denoted as $\mathcal {M}_1$–$\mathcal {M}_5$, using three training methods (See Table 4). First, we trained three models $\mathcal {M}_1$, $\mathcal {M}_2$, and $\mathcal {M}_3$ using the synthetic versions of the three volumes ${\mathcal {V}}_1$–${\mathcal {V}}_3$, respectively. Next, we transfer the weights from $\mathcal {M}_3$ and continue training on a limited number of actual microscopy subvolumes of ${\mathcal {V}}_4$ to produce the fourth model $\mathcal {M}_4$. Finally, we directly train $\mathcal {M}_5$ on subvolumes of actual microscopy data ${\mathcal {V}}_5$ only. After training, we evaluated the NISNet3D-slim models $\mathcal {M}_1$–$\mathcal {M}_5$ using two different evaluation schemes. This is summarized in Supplementary Figure S3. In one scheme we directly test the models $\mathcal {M}_1$, $\mathcal {M}_2$, $\mathcal {M}_4$, and $\mathcal {M}_5$ using all the subvolumes of the actual microscopy volumes ${\mathcal {V}}_1$, ${\mathcal {V}}_2$, ${\mathcal {V}}_4$, and ${\mathcal {V}}_5$, respectively, since they were not used for training. In the case of $\mathcal {M}_3$, we first train $\mathcal {M}_3$ on 50 volumes of synthetic ${\mathcal {V}}_3$ to obtain a pre-trained $\mathcal {M}_3^{\textrm{pre}}$.

For the second scheme we use 3-fold cross-validation to lightly retrain $\mathcal {M}_3^{\textrm{pre}}$ to obtain the final $\mathcal {M}_3$. Specifically, we randomly shuffled 9 subvolumes of ${\mathcal {V}}_3$ and divided them into 3 equal sets where each set contains 3 subvolumes, and then iteratively lightly retrain the $\mathcal {M}_3^{\textrm{pre}}$ on one of the sets and test on the other two sets. We use the average of the evaluation results from the three iterations. We used cross-validation to show the effectiveness of our method when the evaluation data is limited. Note that when lightly retraining $\mathcal {M}_3^{\textrm{pre}}$ we update all its parameters while continue to train on actual microscopy volumes. The training and evaluation scheme for all models is provided in Table 4.

We then investigated how all the methods work when there is no ground truth data for training. For this we train NISNet3D, now known as NISNet3D-synth for these experiments. NISNet3D-synth, $\mathcal {M}_{\textrm{syn}}$, was trained on 950 synthetic microscopy subvolumes that include the synthetic versions of ${\mathcal {V}}_1$–${\mathcal {V}}_4$. Our experience has been that we need more than 800 synthetic microscopy subvolumes for training the types of 3D volumes described in Table 1. We then tested $\mathcal {M}_{\textrm{syn}}$ separately on each manually annotated original microscopy datasets described in Table 1. A figure summarizing the training scheme of NISNet3D-synth and NISNet3D-slim can be found in Supplementary Figure S3.

**Table 5:** Parameters used for NISNet3D nuclei instance segmentation.

Parameters	NISNet3D-slim					NISNet3D-synth			
	${\mathcal {V}}_1$	${\mathcal {V}}_2$	${\mathcal {V}}_3$	${\mathcal {V}}_4$	${\mathcal {V}}_5$	${\mathcal {V}}_1$	${\mathcal {V}}_2$	${\mathcal {V}}_3$	${\mathcal {V}}_4$
$T_{\text{m}}$	5	5	1	1	0	0	0	4	0
$T_{\text{c}}$	700	3000	2000	2000	2000	2000	2000	2000	2000
$T_{\text{{f}}}$	200	500	700	300	200	200	200	500	200

Both NISNet3D-slim and NISNet3D-synth were trained for 100 epochs using the Adam optimizer^77^ with a constant learning rate of 0.001. The weight coefficients of the loss function were set to $\lambda _3=1$, $\lambda _4=10$, $\lambda _5=10$ based on the settings in SpCycleGAN^7,78^, and the hyper-parameters $\alpha _1$, $\alpha _2$ in $\mathcal {L}_{\textrm{TL}}$ were set to 0.3 and 0.7 based on the highest performance configuration^70^, and the hyper-parameters $\beta$, $\gamma$ of $\mathcal {L}_{\textrm{FL}}$^79^ were set to 0.8 and 2 to minimize the training losses. The nuclei instance segmentation parameters used in our experiments are provided in Table 5. These parameters controls how well they split two touching nuclei. Lower $T_{\text{m}}$ will split touching nuclei better. If nuclei size is smaller we should set smaller $T_{\text{c}}$ and $T_{\text{f}}$. We choose these parameters based on the feedback of segmentation qualities (the ability to split touching nuclei visually) from biologist.

### Evaluation metrics

We use object-based metrics to evaluate nuclei instance segmentation accuracy. We define $N_{\textrm{TP}}^t$ as the number of True Positive detections when the Intersection-over-Union (IoU)^80^ between a detected nucleus and a ground truth nucleus is greater than some threshold of *t*. Similarly, $N_{\textrm{FP}}^t$ is the number of False Positives greater than *t*, and $N_{\textrm{FN}}^t$ is the number of False Negatives^81,82^ greater than *t*, respectively. $N_{\textrm{TP}}^t$ measures how many nuclei in a volume are correctly detected, and the higher the value of $N_{\textrm{TP}}^t$ the more accurate the detection. $N_{\textrm{FP}}^t$ represents the detected nuclei that are not actually nuclei but are false detections, and $N_{\textrm{FN}}^t$ represents the number of nuclei that were not detected. A precise and accurate detection method should have high $N_{\textrm{TP}}^t$ but low $N_{\textrm{FP}}^t$ and $N_{\textrm{FN}}^t$ values.

Based on $N_{\textrm{TP}}^t$, $N_{\textrm{FP}}^t$, and $N_{\textrm{FN}}^t$ we define the following metrics that are used to reduce bias^56^: mean Precision ($\textrm{mP}=\frac{1}{|T_{\textrm{IoUs}}|}\sum _{t\in T_{\textrm{IoUs}}}{\frac{N_{\textrm{TP}}^t}{N_{\textrm{TP}}^t+N_{\textrm{FP}}^t}}$), mean Recall ($\textrm{mR}= \frac{1}{|T_{\textrm{IoUs}}|}\sum _{t\in T_{\textrm{IoUs}}}{\frac{N_{\textrm{TP}}^t}{N_{\textrm{TP}}^t+N_{\textrm{FN}}^t}}$), and mean $\mathrm {F_1}$ score ($\mathrm {mF_1}= \frac{1}{|T_{\textrm{IoUs}}|}\sum _{t\in T_{\textrm{IoUs}}}{\frac{2N_{\textrm{TP}}^t}{2N_{\textrm{TP}}^t+N_{\textrm{FP}}^t+N_{\textrm{FN}}^t}}$) that are the mean of Precision, Recall, and the $\mathrm {F_1}$ score, respectively, over a set of multiple IoU thresholds $T_{\textrm{IoUs}}$. We set $T_{\textrm{IoUs}}= \{0.25,0.3,\ldots ,0.45\}$ for datasets ${\mathcal {V}}_1$–${\mathcal {V}}_4$, and set $T_{\textrm{IoUs}}= \{0.5,0.55,\ldots ,0.75\}$ for dataset ${\mathcal {V}}_5$. In addition, we obtained the Average Precision (AP)^61,62^ (a commonly used object detection metric), by estimating the area under the Precision-Recall Curve^63^, using the same sets of thresholds in $T_{\textrm{IoUs}}$. For example, $\textrm{AP}_{.25}$ is the average precision evaluated at an IoU threshold of 0.25. The mean Average Precision (mAP) is then obtained as $\textrm{mAP} = \frac{1}{|T_{\textrm{IoUs}}|}\sum _{t\in T_{\textrm{IoUs}}}{\textrm{AP}_t}$.

The reason for using different thresholds $T_{\textrm{IoUs}}$ for ${\mathcal {V}}_1$–${\mathcal {V}}_4$ compared to those used for ${\mathcal {V}}_5$ is that throughout the experiments we observed that the nuclei in datasets ${\mathcal {V}}_1$–${\mathcal {V}}_4$ are more challenging to segment than the nuclei in ${\mathcal {V}}_5$. Using the same IoU thresholds for evaluating all the datasets resulted in a lower evaluation accuracy for the volumes ${\mathcal {V}}_1$–${\mathcal {V}}_4$ than for ${\mathcal {V}}_5$. Thus, we chose two different sets of IoU thresholds for ${\mathcal {V}}_1$–${\mathcal {V}}_4$, and ${\mathcal {V}}_5$, respectively.

Finally, we use the Aggregated Jaccard Index (AJI)^55^ to integrate object and voxel errors. The AJI is defined as:

12 $$\begin{aligned} \textrm{AJI}=\frac{\sum _{i=1}^{N}{|G_i\cup S_m^i|}}{\sum _{i=1}^{N}{|G_i\cap S_m^i|}+\sum _{j\in U}{|S_j|}} \end{aligned}$$

where $G_i$ denotes the *i*th nucleus in the ground truth volume *G* having a total number of *N* nuclei, *U* is the volume of segmented nuclei without corresponding ground truth, and $S_m^i$ is the *m*th connected component in the segmentation mask which has the largest Jaccard Index/similarity with $G_i$. Note that each segmented nucleus with index *m* that belongs to the *m*th connected component cannot be used more than once.

The values of all the parameters for each method were chosen to achieve the best visual results, as discussed in “Discussion”. The values of the various metrics used are given in Tables 2 and 3 for each microscopy dataset. Figure 6 shows the AP scores using multiple IoU thresholds for each subvolume of the dataset ${\mathcal {V}}_1$–${\mathcal {V}}_5$. The orthogonal views (*XY* focal planes and *XZ* focal planes) of the segmentation masks are overlaid on the original microscopy subvolume for each method on ${\mathcal {V}}_1$–${\mathcal {V}}_5$ as shown in Fig. 4, Supplementary Figures S4 and S5. Note that the different colors correspond to different nuclei.

### Test microscopy volumes and ground truth annotations

We used four different actual microscopy 3D volumes in our experiments, denoted by ${\mathcal {V}}_1$–${\mathcal {V}}_4$, having fluorescent-labeled (DAPI or Hoechst 33342 stain) nuclei that were collected from cleared rat kidneys (Scale^37^ and BABB^38^), rat livers, and cleared mouse intestines using confocal microscopy. These volumes were used to demonstrate the performance of NISNet3D, to generate training data for NISNet3D-slim, and to generate annotations that provide the ground truth for quantitative evaluation of NISNet3D and other segmentation methods. ${\mathcal {V}}_1$ differs from ${\mathcal {V}}_3$ in that ${\mathcal {V}}_1$ includes fluorescent objects that are not nuclei, and thus must be distinguished from nuclei^37^. In addition, we also used a publicly available electron microscopy zebrafish brain volume, ${\mathcal {V}}_5$, known as NucMM^83^. We used the NucMM dataset and its provided ground truth to demonstrate that NISNet3D not only works on fluorescent microscopy volumes but also works with other high resolution imaging modalities.

## Supplementary Information

Supplementary Information.

## Data Availability

The datasets analyzed during the current study are available in the Zenodo repository https://doi.org//10.5281/zenodo.7065147^39^.

## Code availibility

The NISNet3D source code package is available at https://gitlab.com/viper-purdue/nisnet-release. The source code is released under Creative Commons License Attribution-NonCommercial-ShareAlike-CC BY-NC-SA. The source code cannot be used for commercial purposes. Address all correspondence to Edward J. Delp, ace@ecn.purdue.edu.
