# Supplementary material for: NISNet3D: three-dimensional nuclear synthesis and instance segmentation for fluorescence microscopy images
Source: Sci Rep. 2023 Jun 12;13:9533. doi: 10.1038/s41598-023-36243-9 (PMC10261124; doi:10.1038/s41598-023-36243-9)
Supplement: Supplementary file 1 — Supplementary Information. [file 41598_2023_36243_MOESM1_ESM.pdf]

## Supplementary Material

**Supplementary Table S1.** Comparison of voxel-based evaluation metrics for microscopy datasets $\mathscr{V}_1$ - $\mathscr{V}_4$. Scores range from 0 to 1, with closer to 1 being better. The best performance with respect to each metric are in bold. The methods above the double line are results using both synthetic and actual microscopy volumes for training, and use actual volumes for testing. The methods below the double line use only synthetic data for training but use actual volumes for testing.

| Methods | Microscopy $\mathscr{V}_1$ | | Microscopy $\mathscr{V}_2$ | | Microscopy $\mathscr{V}_3$ | | Microscopy $\mathscr{V}_4$ | |
|---|---|---|---|---|---|---|---|---|
| | Dice | IoU | Dice | IoU | Dice | IoU | Dice | IoU |
| **Methods using partially annotated actual volumes and synthetic volumes** | | | | | | | | |
| 3D Watershed[46] | 0.6453 | 0.4764 | 0.8103 | 0.6843 | 0.7918 | 0.6582 | 0.7396 | 0.5939 |
| Squassh[47] | 0.5292 | 0.3598 | **0.8357** | **0.7219** | 0.7907 | 0.6592 | 0.7483 | 0.5991 |
| CellProfiler[48] | 0.6009 | 0.4295 | 0.7877 | 0.6572 | 0.7694 | 0.6335 | 0.6575 | 0.4990 |
| VTEA[54] | 0.5505 | 0.3798 | 0.7134 | 0.5579 | 0.7001 | 0.5440 | 0.7172 | 0.5609 |
| VNet[24] | 0.7439 | 0.5922 | 0.7023 | 0.5436 | 0.7929 | 0.6614 | 0.6964 | 0.5402 |
| 3D U-Net[23] | 0.7663 | 0.6211 | 0.7336 | 0.5803 | **0.8259** | **0.7056** | 0.6984 | 0.5429 |
| Cellpose[3] | 0.7618 | 0.6153 | 0.7395 | 0.5877 | 0.7941 | 0.6601 | 0.6822 | 0.5193 |
| DeepSynth[6] | **0.8182** | **0.6924** | 0.7442 | 0.5937 | 0.8038 | 0.6785 | 0.6691 | 0.5030 |
| **NISNet3D-slim** | 0.7557 | 0.6073 | 0.7676 | 0.6243 | 0.8135 | 0.6872 | **0.7993** | **0.6661** |
| **Methods trained using synthetic volumes but tested on actual volumes** | | | | | | | | |
| Cellpose-synth[3] | **0.7972** | **0.6628** | **0.8569** | **0.7508** | **0.8209** | **0.6971** | 0.7160 | 0.5585 |
| StarDist3D-synth[15] | 0.7030 | 0.5420 | 0.7251 | 0.5721 | 0.7314 | 0.5773 | 0.6723 | 0.5081 |
| nnU-Net-synth[45] | 0.7476 | 0.5969 | 0.7994 | 0.6674 | 0.8043 | 0.6749 | 0.7520 | **0.6082** |
| **NISNet3D-synth** | 0.7679 | 0.6232 | 0.8352 | 0.7190 | 0.8058 | 0.6761 | **0.7526** | 0.6052 |

Supplementary Table S1 shows the comparison of voxel-based evaluation metrics for microscopy datasets $\mathscr{V}_1$-$\mathscr{V}_4$. NISNet3D, Cellpose[3], StarDist[15], nnU-Net[45], VNet[24], and 3D U-Net[23] all have similar Dice and IoU scores and outperform the other methods.

NISNet3D is designed for instance segmentation which means how accurate it splits touching nuclei is more relevant than the voxel-based semantic segmentation of the entire foreground and background. Please refer back to Table 2 for the object-based metrics for instance segmentation.

**Supplementary Table S2.** Comparison of voxel-based evaluation metrics for microscopy dataset $\mathscr{V}_5$. Scores range from 0 to 1, with closer to 1 being better. The best performance with respect to each metric are in bold.

| Methods | Microscopy $\mathscr{V}_5$ | |
|---|---|---|
| | Dice | IoU |
| StarDist3D[15] | 0.8285 | 0.7076 |
| DeepSynth[6] | 0.8849 | 0.7946 |
| Cellpose[3] | 0.9015 | 0.8351 |
| **NISNet3D-slim** | **0.9279** | **0.8664** |

Supplementary Table S2 shows the comparison of voxel-based evaluation metrics for microscopy dataset $\mathscr{V}_5$. NISNet3D does have the highest Dice[84] and IoU scores for this particular volume. Both DeepSynth[6] and Cellpose[3] have slightly lower scores, but StarDist3D[15] shows much lower Dice and IoU.

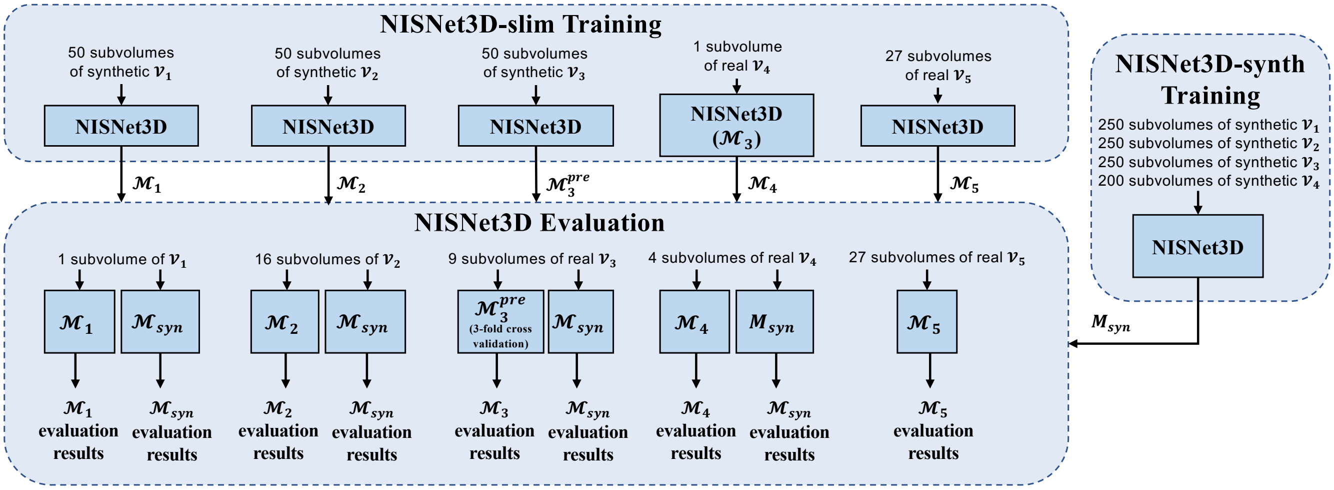


**Supplementary Figure S3.** Training Scheme of NISNet3D-synth and NISNet3D-slim

Supplementary Figure S3 is a summary of the training scheme mention in Section 4.5. As mentioned above, we provide two versions of NISNet3D: NISNet3D-slim (including 5 models $\mathscr{M}_1$-$\mathscr{M}_5$) and NISNet3D-syn (including 1 model $\mathscr{M}_{\text{syn}}$) for different application scenarios. We trained 5 versions of NISNet3D-slim, denoted as $\mathscr{M}_1$-$\mathscr{M}_5$, using three training methods (See Table 4). First, we trained three models $\mathscr{M}_1$, $\mathscr{M}_2$, and $\mathscr{M}_3$ using the synthetic versions of the three volumes $\mathscr{V}_1$-$\mathscr{V}_3$, respectively. Next, we transfer the weights from $\mathscr{M}_3$ and continue training on a limited number of actual microscopy subvolumes of $\mathscr{V}_4$ to produce the fourth model $\mathscr{M}_4$. Finally, we directly train $\mathscr{M}_5$ on subvolumes of actual microscopy data $\mathscr{V}_5$ only. After training, we evaluate the models $\mathscr{M}_1$-$\mathscr{M}_5$ through two different evaluation schemes. In one method we directly test the models $\mathscr{M}_1$, $\mathscr{M}_2$, $\mathscr{M}_4$, and $\mathscr{M}_5$ using all the subvolumes of the actual microscopy volumes $\mathscr{V}_1$, $\mathscr{V}_2$, $\mathscr{V}_4$, and $\mathscr{V}_5$, respectively, since they were not used for training. In the case of $\mathscr{M}_3$, we first train $\mathscr{M}_3$ on 50 volumes of synthetic $\mathscr{V}_3$ to obtain a pre-trained $\mathscr{M}_3^{\text{pre}}$. Then we use 3-fold cross-validation to continue lightly retrain $\mathscr{M}_3^{\text{pre}}$ to obtain the final $\mathscr{M}_3$. Specifically, we randomly shuffled 9 subvolumes of $\mathscr{V}_3$ and divided them into 3 equal sets where each set contains 3 subvolumes, and then iteratively lightly retrain the $\mathscr{M}_3^{\text{pre}}$ on one of the sets and test on the other two sets. We use the average of the evaluation results from the three iterations. We used cross-validation to show the effectiveness of our method when the evaluation data is limited. Note that when lightly retraining $\mathscr{M}_3^{\text{pre}}$ we update all its parameters while continue to train on actual microscopy volumes. The training and evaluation scheme for all models is provided in Table 4. In addition, to demonstrate that our method works even there is no ground truth data for training, we provide another version of NISNet3D called NISNet3D-synth which includes 1 model $\mathscr{M}_{\text{syn}}$ was trained on 950 synthetic microscopy subvolumes that include the synthetic versions of $\mathscr{V}_1$-$\mathscr{V}_4$.

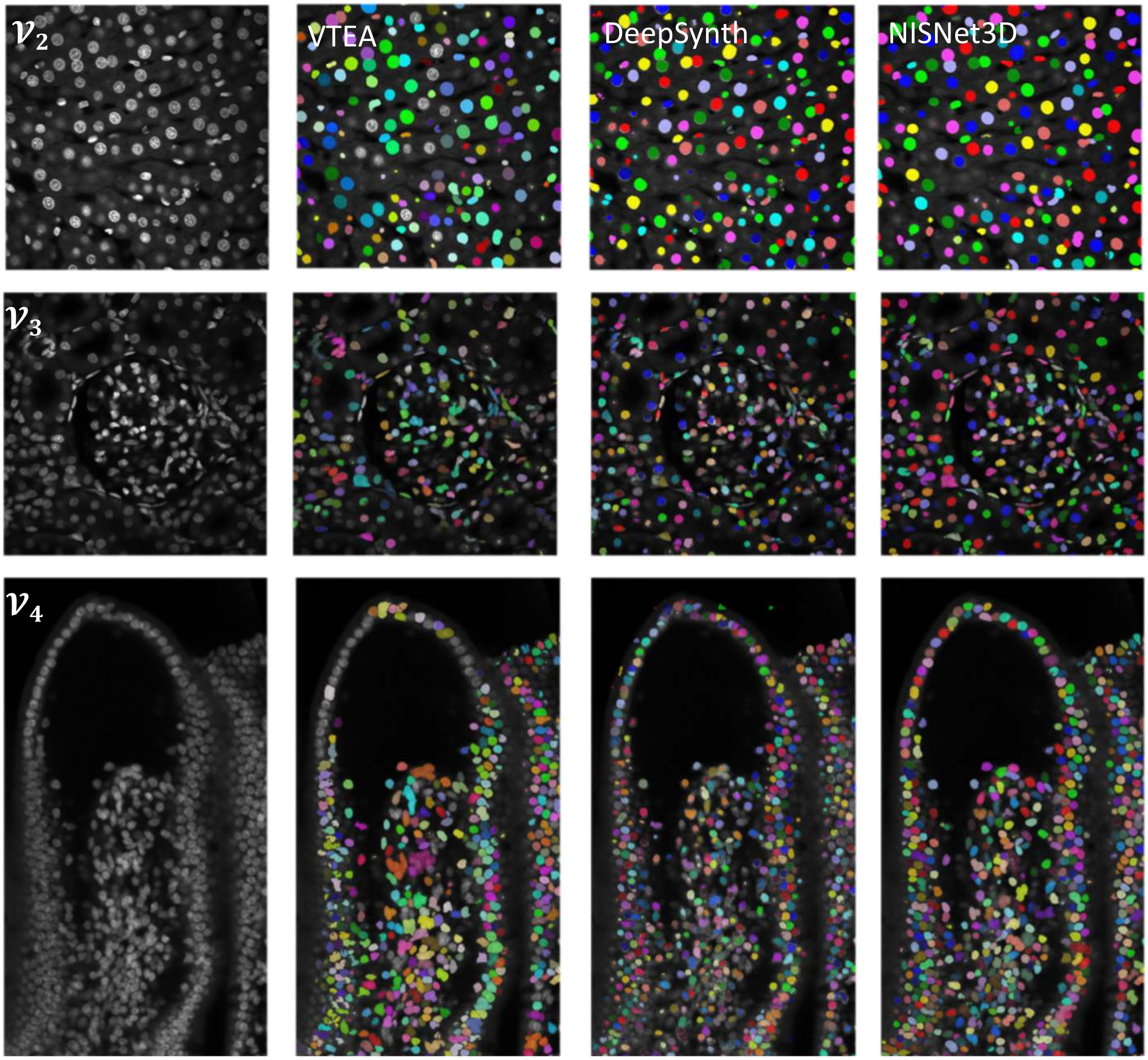


**Supplementary Figure S4.** Three volumes of fluorescent-labeled nuclei $\mathscr{V}_2$, $\mathscr{V}_3$, $\mathscr{V}_4$ were segemented in 3D by three approaches. The deep learning approaches perform semantic segmentation with similar efficacy. DeepSynth over-segments nuclei, compared to NISNet3D suggesting NISNet3D improves instance segmentation.

Supplementary Figure S4 and Supplementary Figure S5 are additional figures that show the qualitative performance of NISNet3D segmentation.

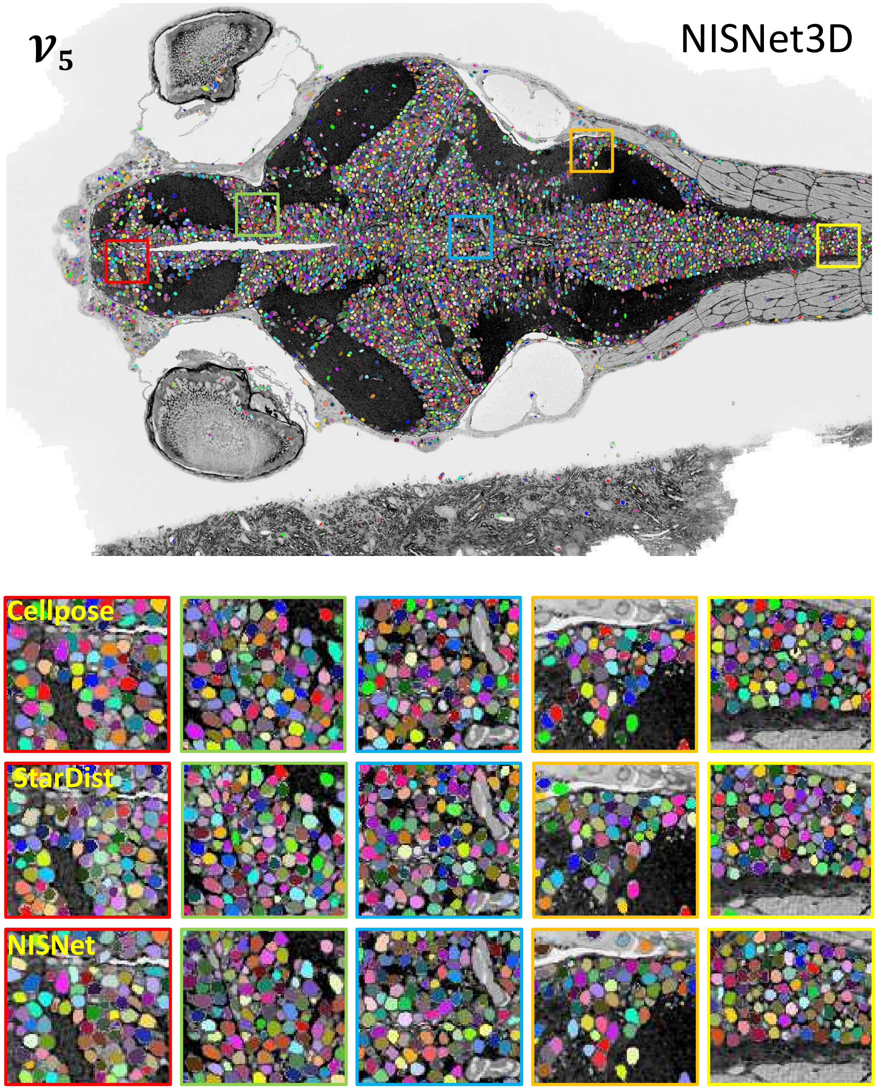


**Supplementary Figure S5.** NISNet3D segmentation of a 3D electron microscopy volume ($\mathcal{V}_5$) demonstrates multimodal utility with comparable accuracy to other segmentation approaches. Select regions segmented in this volume are indicated in the top panel and insets are provided below. We use different colors to distinguish different nuclei instances segmented by corresponding methods
